# Grid cells accurately track movement during path integration-based navigation despite switching reference frames

**DOI:** 10.1038/s41593-025-02054-6

**Published:** 2025-09-10

**Authors:** Jing-Jie Peng, Beate Throm, Maryam Najafian Jazi, Ting-Yun Yen, Rocco Pizzarelli, Hannah Monyer, Kevin Allen

**Affiliations:** 1 Medical Faculty of Heidelberg University and German Cancer Research Center, Heidelberg, Germany; 2 EBRI, Rome, Italy

**Keywords:** Neural circuits, Neural decoding, Spatial memory

## Abstract

Grid cells, with their periodic firing fields, are fundamental units in neural networks that perform path integration. It is widely assumed that grid cells encode movement in a single, global reference frame. In this study, by recording grid cell activity in mice performing a self-motion-based navigation task, we discovered that grid cells did not have a stable grid pattern during the task. Instead, grid cells track the animal movement in multiple reference frames within single trials. Specifically, grid cells reanchor to a task-relevant object through a translation of the grid pattern. Additionally, the internal representation of movement direction in grid cells drifted during self-motion navigation, and this drift predicted the mouse’s homing direction. Our findings reveal that grid cells do not operate as a global positioning system but rather estimate position within multiple local reference frames.

## Abstract (web summary)

Grid cells do not maintain a stable pattern during a self-motion-based task, but track animal movement in multiple local reference frames and reanchor to task-relevant objects, thus estimating local rather than global position.

## Main

Path integration is a cognitive process whereby an animal estimates its movement from self-motion cues to maintain an up-to-date representation of its position and orientation^1–4^. This process is active whenever an animal moves and contributes to forming new cognitive maps^2,5^. In environments with salient landmarks, animals use a combination of path integration and landmark-based strategies to keep track of their position^6,7^. When landmarks are absent or not readily visible, the position and orientation of an animal are estimated primarily from path integration. Due to error accumulation^8–13^, the quality of these estimates degrades over time, making navigation using path integration alone more suited for short navigation journeys.

Lesion studies show that the medial entorhinal cortex (MEC) contributes to the ability of an animal to navigate using path integration^13–16^. However, the activity patterns of neurons within this brain region in freely moving animals during the path integration process have yet to be established. The functional cell type within this brain area that is most often linked to path integration is the grid cell^3,17–23^. In randomly foraging animals, grid cells have several firing fields organized as one continuous grid of equilateral triangles. It has been proposed that grid cells provide a global coordinate system in which the current location and orientation of an animal are updated via self-motion cues^3,24–27^. However, it is still unclear whether the continuous grid firing pattern of grid cells is present in animals navigating two-dimensional (2D) environments primarily by path integration, as this grid pattern has been observed mainly in animals performing random foraging tasks in open-field environments or navigation tasks with salient landmarks^21,28,29^.

Here, we set out to characterize the firing patterns of grid cells in mice navigating using path integration and tested whether grid cell activity predicted homing behavior. We took advantage of the newly established AutoPI homing task, which allows large-scale electrophysiological recordings of spatially selective neurons in mice navigating a 2D environment utilizing path integration^30^. Moreover, we developed a deep-learning decoding framework to monitor the path integration process in grid cell modules with high temporal resolution and showed that grid map orientation predicts homing behavior.

## Results

### Mice performing the AutoPI task use path integration

**Fig. 1:**
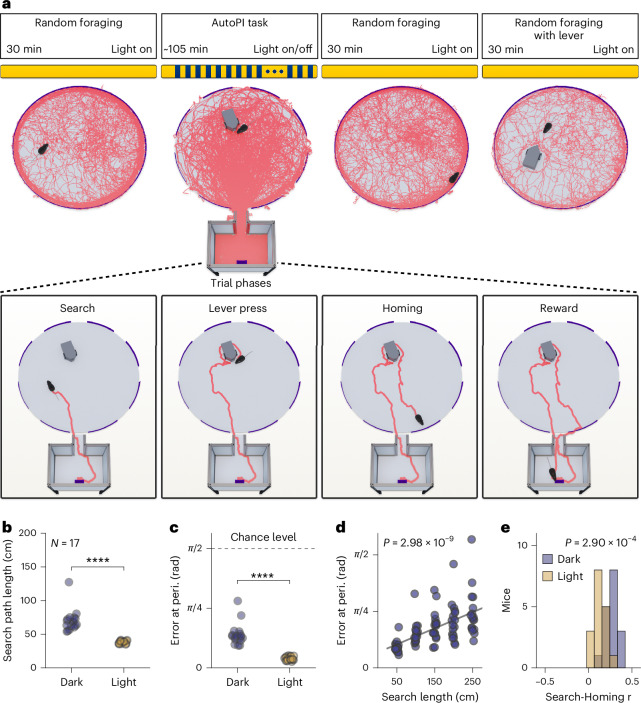
Recording protocol and behavioral performance of mice on the AutoPI task. **a**, Top, Recording protocol. During each recording session, mice performed a random foraging task before and after the AutoPI task. On the AutoPI task, mice alternated between trials with and without visible light (light and dark trials). In the last recording session, mice performed an additional random foraging trial in which the lever box was placed on the arena. Random foraging with the lever box placed on the arena consists of two recordings of 15 min each, and the lever is placed at a different location after the first 15-min recording. The red lines on the arena and floor of the home base are examples of the path of a mouse during a recording session. Bottom, The four phases of a trial on the AutoPI task. The mouse leaves the home base and searches for the lever box on the arena. Once at the lever box, the lever is pressed, triggering food reward delivery in the food magazine of the home base. The mouse returns to the home base (homing) to access the food reward. **b**, Search path length during dark and light trials (dark–light difference: *N* = 17 mice, two-sided Wilcoxon signed-rank test, statistic = 0.0, *P* = 1.53 × 10^−5^). **c**, Homing error at the periphery during dark and light trials. For definitions, see Extended Data Fig. 2a (*N* = 17 mice, two-sided Wilcoxon signed-rank test, statistic = 0.0, *P* = 1.53 × 10^−5^). **d**, Homing error as a function of search path length during dark trials (*N* = 17 mice, two-sided Friedman test, statistic = 45.6, *P* = 2.98 × 10^−9^). **e**, Pearson correlation coefficients between search path length and homing error during light and dark trials (*N* = 17 mice, difference between light and dark trials, two-sided Wilcoxon signed-rank test, statistic = 7.0, *P* = 2.90 × 10^−4^). ****P < 0.0001. Source data

**Extended Data Fig. 1:**
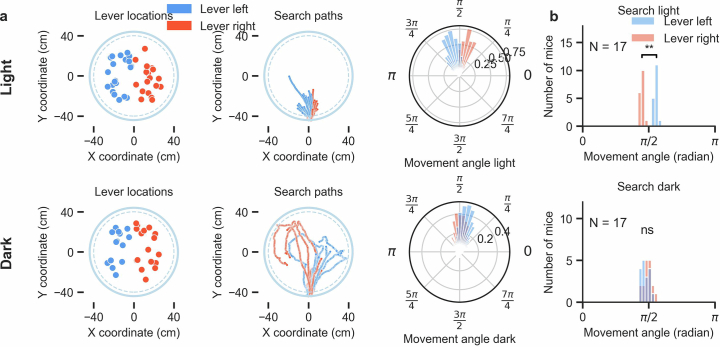
Distinct search behavior in dark versus light conditions. **a**, Left: Lever location in one session during light (top) and dark (bottom) trials. Trials are divided into two groups depending on whether the lever was on the left (blue) or right (red) side of the arena. Units of the graph are in centimeters, (0,0) corresponds to the center of the arena. Second column: First half of the search path in the same session during light (top) and dark (bottom) trials. Paths are shown in blue and red for trials in which the lever was on the left and right side of the arena, respectively. Note that the path does not end at the lever location because only the first half of the search path is shown. Third column: Distribution of movement heading during the first half of search during the same recording session. Blue and red correspond to trials when the lever was on the left and right side of the arena, respectively. **b**, Distribution of median movement heading during the first half of the search during light (top) and dark (bottom) trials, for trials where the lever was on the left (blue) or right (red) of the arena (N = 17 mice, two-sided Kuiper two-sample test, Light: statistic = 289, *P* = 0.001; Dark: statistic = 85, *P* = 1.0). ** 0.001 < *P* < 0.01. Source data

**Extended Data Fig. 2:**
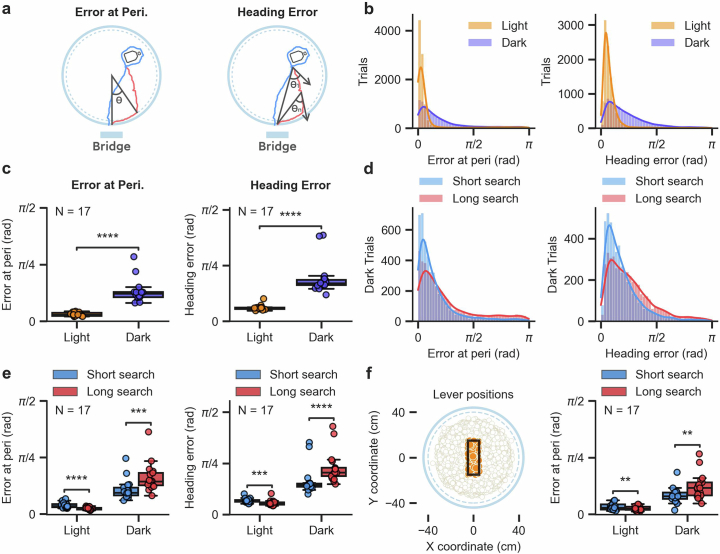
Error at the periphery and heading error of the mouse in different light and search conditions. **a**, Illustration of two measures of homing error: error at the periphery (left) and heading error. Error at the periphery is the angle between the animal’s position when it reaches the periphery of the arena after pressing the lever, the center of the arena, and the center of the bridge. Heading error is calculated along the homing path (at 50 Hz) and is the angle between two vectors with origin at the animal’s position, one pointing in the animal’s direction of movement and the other pointing towards the bridge. The median heading error is calculated for each trial. **b**, Distribution of the error at the periphery (left) and the heading error (right) for homing paths in light and dark trials. **c**, Median error at the periphery (left) or median heading error (right) for each mouse in light and dark trials (N = 17 mice, two-sided Wilcoxon signed-rank test, error at the periphery: *P* = 1.526 ×10^−5^, heading error: *P* = 1.526 ×10^−5^). Box plots show median (center line), first and third quartiles (box bounds), and 1.5 × interquartile range (whiskers). **d**, Distribution of the error at the periphery (left) and heading error (right) for dark trials with short and long search paths. Trials within a session were divided into short and long search paths depending on whether they were below or above the median search path length of the session. **e**, Median error at the periphery (left) or heading error (right) for each mouse during light and dark trials with short and long search paths (N = 17 mice, two-sided Wilcoxon signed-rank test, error at the periphery, light: *P* = 1.526 ×10^−5^, dark: *P* = 2.136 ×10^−4^; heading error, light: P = 6.561 ×10^−4^, dark: *P* = 1.526 ×10^−5^). Box plots show median (center line), first and third quartiles (box bounds), and 1.5 × interquartile range (whiskers). **f**, Effect of search path length during trials in which the lever was located near the center of the arena. We considered only trials in which the lever was located within a 10 ×30 cm rectangle centered on the arena center (left). Error at the periphery for each mouse for long and short search paths in light and dark trials (right, N = 17 mice, two-sided Wilcoxon signed-rank test, error at the periphery, light: *P* = 5.569 ×10^−3^, dark: *P* = 6.653 ×10^−3^). Box plots show median (center line), first and third quartiles (box bounds), and 1.5 × interquartile range (whiskers). ****P < 0.0001, ***P < 0.001, ** 0.001 < *P* < 0.01. Source data

To explore the firing activity patterns of MEC neurons during path integration behavior, 17 mice were trained on the AutoPI task and implanted with silicon probes targeted at the superficial layers of MEC. The spike activity and the behavior were recorded during random foraging trials and while the mice performed the AutoPI task (Fig. 1a). The AutoPI apparatus consisted of an arena connected to a home base via a bridge. In each trial of the AutoPI task, the mouse left the home base, searched for a randomly located lever box on the arena, and pressed the lever. Pressing the lever triggered the release of a food reward in the food magazine of the home base. The mouse then returned to the home base (homing) to collect the food reward. The mouse performed AutoPI trials under normal lighting conditions (light trials) and in complete darkness (dark trials). Every session started with seven consecutive light trials followed by an alternating sequence of dark and light trials. The arena was rotated after each trial to one of eight possible angles (multiple of 45°). The lever moved to a new random location on the arena every fourth trial. The combination of arena rotations and lever movement ensured that the lever moved to a new location relative to the home base in most trials. The last session for each mouse was concluded with a random foraging trial with the lever in two different positions on the arena to account for a potential influence of the lever on the grid cell activity (Fig. 1a). A total of 180 sessions (ranging from 3 to 25 sessions per mouse, median of 10 sessions) with 38 to 172 trials per session (median of 112 trials) were recorded. When comparing the behavior between light and dark trials, we found that mice had longer search paths to the lever during dark trials (Fig. 1b). In dark trials, we found no evidence that mice could perceive the lever’s location before reaching it (Extended Data Fig. 1). To assess the ability of the mice to return directly to the home base after pressing the lever, we defined the error at the periphery as the angle between two vectors with their origin at the center of the arena and pointing to the bridge of the home base and to the location at which the mouse reached the periphery of the arena, respectively (Extended Data Fig. 2a). Error at the periphery during dark trials was larger than during light trials (Fig. 1c and Extended Data Fig. 2b,c). Still, it remained above the chance level (chance level = π/2), demonstrating that although mice made larger homing errors in darkness, they preferentially headed toward the home base.

**Extended Data Fig. 3:**
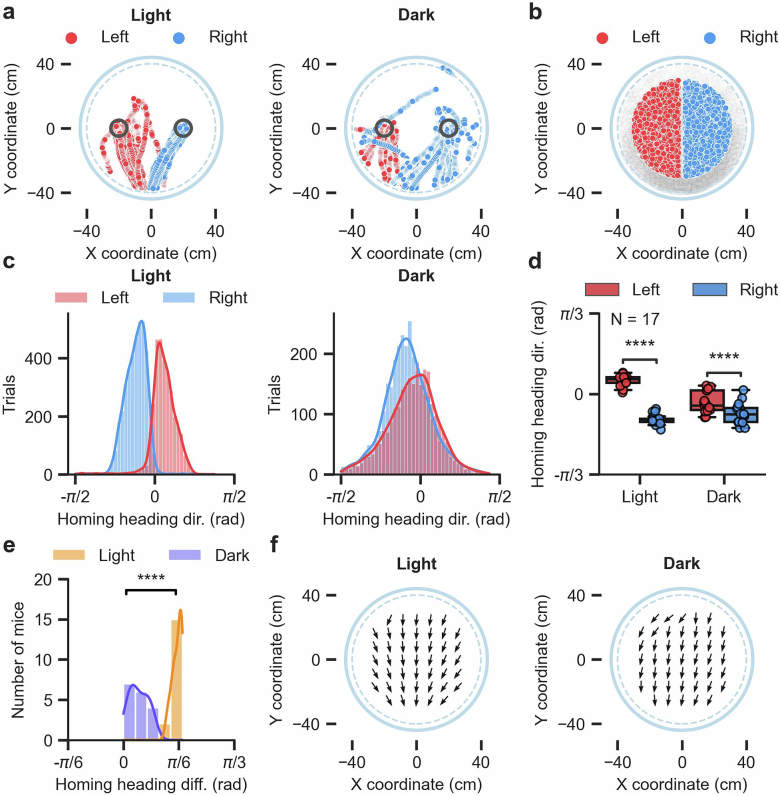
Homing heading direction of the mouse as a function of the animal’s position at the start of the homing path. **a**, Examples of homing paths from one mouse starting at two distinct locations on the arena (two black circles). The homing paths of light and dark trials are shown separately. The arena border is depicted as a gray dashed circle. **b**, Starting position of homing paths grouped based on whether the starting position was on the left or right side of the arena. We excluded starting positions that were more than 30 cm away from the arena center. Starting positions within a 2-cm-wide vertical band at the center of the arena were also excluded. **c**, Distribution of median homing heading direction for homing paths starting on the left or right side of the arena. Data are shown separately for light and dark trials. **d**, Median homing heading direction for each mouse for homing paths starting on the left or right side of the arena (N = 17 mice, two-sided Wilcoxon signed-rank test, light trials: *P* = 1.526 ×10^−5^, Dark trials: *P* = 3.052 ×10^−5^). Box plots show median (center line), first and third quartiles (box bounds), and 1.5 × interquartile range (whiskers). **e**, Difference in median homing direction for homing path starting on the left or right side of the arena. One data point per mouse. The data are shown separately for light and dark trials (N = 17 mice, two-sided Wilcoxon signed-rank test, *P* = 1.526 ×10^−5^). **f**, Vector field of the median homing direction of homing paths starting at different positions on the arena. Each arrow represents the median homing direction of homing paths starting at the location of the arrow. The data from all mice are included and shown separately for light and dark trials. ****P < 0.0001. Source data

When mice perform path integration, the error in the internal representation of space increases over the distance covered^2,31,32^. Indeed, we found a linear correlation between search path length and homing error (Fig. 1d). This correlation was not dependent on the lateral position of the lever on the arena (Extended Data Fig. 2d–f). The correlation coefficients between the homing error and search path length calculated for each mouse were larger during dark trials than in light trials (Fig. 1e). We also observed that the position of the lever on the arena influenced the homing direction in light and dark trials, although this effect was stronger during light than dark trials (Extended Data Fig. 3). Taken together, these results suggest that, while we cannot entirely rule out the usage of external cues, mice primarily used path integration to return to the home base during dark trials.

### Absence of stable grid patterns in mice navigating using self-motion information

**Fig. 2:**
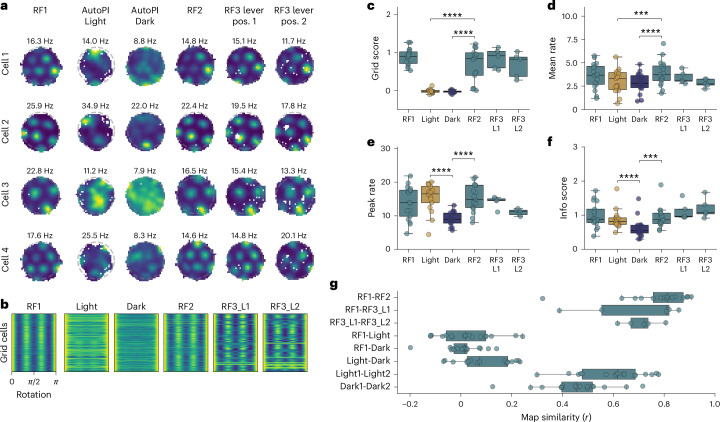
Reduced grid cell periodicity in mice performing a homing task. **a**, Firing rate maps of four grid cells during the different tasks (random foraging (RF) and AutoPi task). RF3 lever position 1 (RF3_L1): random foraging task with the lever on the arena. RF3 lever position 2 (RF3_L2): random foraging task with the lever moved to a different location on the arena. Example cells are from three mice. (cell 1 and 4: mouse 1, cell 2: mouse 2, cell 3: mouse 3). **b**, Grid cell periodicity matrix in different tasks. Each row of the matrix represents the data from one grid cell. Grid periodicity is reflected by the peaks for the rotations of 0, π/3, and 2 × π/3 rad. **c**, Grid scores for grid cells during RF trials and light and dark trials of the AutoPI task (*N* = 17 mice except for RF3 lever where *N* = 5 mice, two-sided Wilcoxon signed-rank test, Light-RF2 difference: statistic = 0.0, *P* = 1.53 × 10^−5^; Dark-RF2 difference: statistic = 0.0, *P* = 1.53 × 10^−5^). Demonstrating reduced grid score during the AutoPI task. **d**, Mean firing rate of grid cells (Two-sided Wilcoxon signed-rank test; Light-RF2: statistic = 8.0, *P* = 3.81 × 10^−4^; Dark-RF2: statistic = 1.0, *P* = 3.05 × 10^−5^; Light-Dark: statistic = 40.0, *P* = 0.089). **e**, Peak firing rate in the firing rate maps of grid cells (Two-sided Wilcoxon signed-rank test; Light-RF2: statistic = 62.0, *P* = 0.52; Dark-RF2: statistic = 1.0, *P* = 3.05 × 10^−5^; Light-Dark: statistic = 1.0, *P* = 3.05 × 10^−5^). **f**, Information scores of grid cells (Two-sided Wilcoxon signed-rank test; Light-RF2: statistic = 45.0, *P* = 0.15; Dark-RF2: statistic = 4.0, *P* = 1.07 × 10^−4^; Light-Dark: statistic = 0.0, *P* = 1.53 × 10^−5^). **g**, Firing rate map similarity of grid cells across different conditions. The correlation coefficient (*r* value) between the firing rate maps are shown. Dots are the medians of individual mice. (*N* = 17 mice apart from the RF3_L1 and RF3_L2 where *N* = 5 mice, two-sided Wilcoxon signed-rank test, RF1-RF2 versus RF1-Light: statistic = 0.0, *P* = 1.53 × 10^−5^; RF1-RF2 versus RF1-Dark: statistic = 0.0, *P* = 1.53 × 10^−5^; RF1-RF3_L1 versus RF3_L1-RF3_L2: statistic = 7, *P* = 1.0; RF1-Light versus Light1-Light2: statistic = 0.0, *P* = 1.53 × 10^−5^; RF1-Dark versus Dark1-Dark2: statistic = 0.0, *P* = 1.53 × 10^−5^; Light1-Light2 versus Dark1-Dark2: statistic = 17.0, *P* = 0.0032; Light-Dark versus Light1-Light2: statistic = 0.0, *P* = 1.53 × 10^−5^; Light-Dark versus Dark1-Dark2: statistic = 0.0, *P* = 1.53 × 10^−5^). Box plots show the median (center line), first and third quartiles (box bounds) and 1.5 times the interquartile range (whiskers). No adjustments were made for multiple comparisons. ****P < 0.0001, ***P < 0.001. Source data

**Extended Data Fig. 4:**
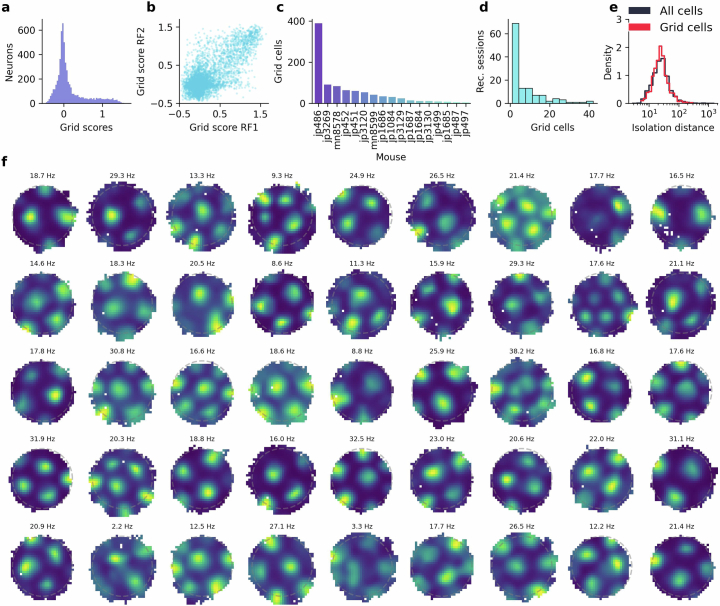
Grid cell identification and number of grid cells per mouse and session. **a**, Distribution of grid scores from all recorded neurons in the first random foraging trials. **b**, Grid scores during the first (RF1) and second (RF2) random foraging trials for all recorded neurons. **c**, Number of grid cells per mouse. **d**, Number of grid cells per recording session. **e**, Distribution of isolation distance of all cells and grid cells. **f**, Firing rate map of a random sample of grid cells with a statistically significant grid score during the first and second random foraging trials. Source data

**Extended Data Fig. 5:**
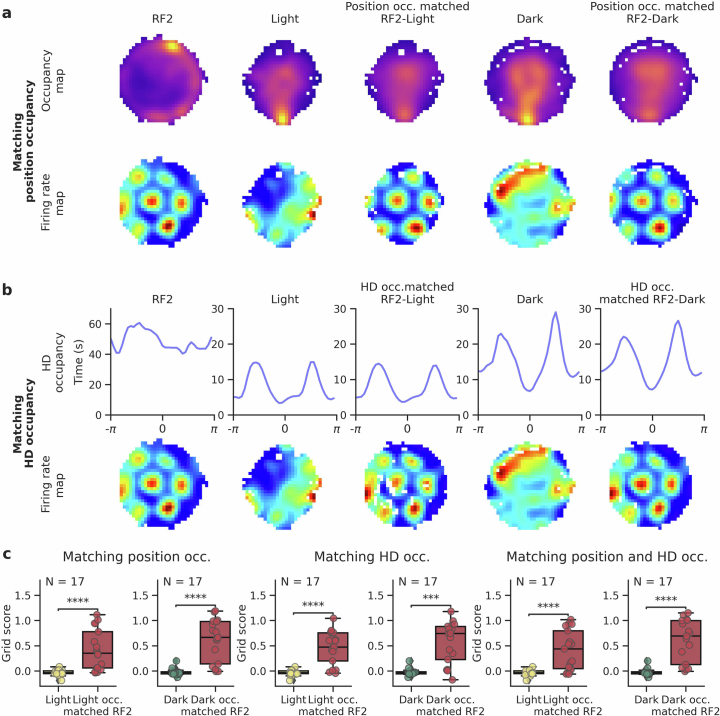
Position and head-direction occupancy during the AutoPI task does not explain degradation in grid-patterned activity. **a**, First row: position occupancy for different conditions. To address the issue of location sampling and occupancy on grid pattern activity, we downsampled position in the second random foraging to match the occupancy map of the AutoPI task light condition (Column 3) or dark condition (Column 5). Second row: firing rate map for different conditions match the occupancy map in the first row. **b**, To address the issue of HD sampling and occupancy, HD in the second random foraging was downsampled to match the HD occupancy of the AutoPI task light condition (Column 3) or dark condition (Column 5). **c**, Grid scores in the RF2 occupancy matched to light or dark conditions are compared to the observed grid scores in the light or dark trials of the AutoPI task. The comparisons include downsampling for position occupancy (N = 17 mice, two-sided Wilcoxon signed-rank test, Light vs. Light occ. matched RF2: *P* = 4.578 ×10^−5^, Dark vs. Dark occ. matched RF2: *P* = 3.052 ×10^−5^), HD occupancy (N = 17 mice, two-sided Wilcoxon signed-rank test, Light vs. Light occ. matched RF2: *P* = 4.578 ×10^−5^, Dark vs. Dark occ. matched RF2: *P* = 1.526 ×10^−4^), and combined adjustments for both factors (N = 17 mice, two-sided Wilcoxon signed-rank test, Light vs. Light occ. matched RF2: *P* = 7.629 ×10^−5^, Dark vs. Dark occ. matched RF2: *P* = 3.052 ×10^−5^). Box plots show median (center line), first and third quartiles (box bounds), and 1.5 × interquartile range (whiskers). ****P < 0.0001, ***P < 0.001. Source data

We recorded 5,746 neurons, of which 931 were classified as grid cells based on their significant grid scores during the first random foraging trial (Extended Data Fig. 4). The standard firing rate maps of grid cells showed spatial periodicity during both random foraging trials but, surprisingly, not during the AutoPI task (Fig. 2a,b). Grid scores were lower during the light and dark trials of the AutoPI task compared to those observed during random foraging trials (Fig. 2c). The decrease in grid periodicity observed during the AutoPI task was not simply the result of the lever being present on the arena. Indeed, when mice performed a random foraging trial in which the lever was on the arena (RF3 lever), grid cells displayed strong periodicity (Fig. 2a–c). Moreover, the decreased grid scores observed during the AutoPI task were not simply due to a reduced spatial coverage or biased head-direction sampling during the task (Extended Data Fig. 5). Grid cells had a slightly lower mean firing rate during the AutoPI task (light trials and dark trials) than during random foraging (Fig. 2d). The peak firing rate and the information score of grid cells were significantly lower during dark trials than light trials of the AutoPI task, which may indicate that the firing fields of grid cells on the arena during dark trials were unstable (Fig. 2e,f).

Given these changes in firing properties of grid cells during the AutoPI task, we tested map similarity between random foraging behavior and the AutoPI task (Fig. 2g). Maps were strongly correlated across the three random foraging trials independently of the presence of the lever on the arena (median RF1-RF2 *r* value = 0.813, median RF1-RF3_L1 of 0.809), but much less when comparing the random foraging trials to the light or dark trials of the AutoPI task (median RF1-Light = 0.017, median RF1-Dark = 0.014). Map similarity between light and dark trials was also strikingly low, suggesting altered spatially selective firing activity. Interestingly, map similarity between two independent sets of light trials was higher than for two independent sets of dark trials, again pointing toward less stable firing fields during dark trials. Still, both were higher than when compared to random foraging, indicating some degree of preserved spatial coherence during the AutoPI task.

### Grid cell firing pattern modulated by lever location on the arena

**Fig. 3:**
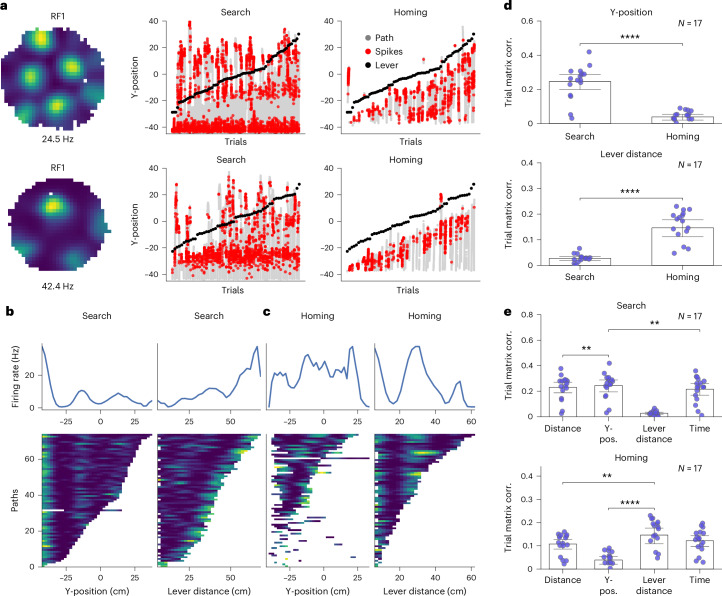
Firing fields of grid cells modulated by lever location in darkness. **a**, Examples of two grid cells. First column: Firing rate maps during random foraging. Second column: Firing of the neuron plotted as a function of the mouse position on the *y* axis of the arena (Y-position, 0 representing the center of the arena and −40 marking the edge near the bridge) during search. All search paths (gray lines) are shown next to each other and ranked according to the Y-position of the lever box (black dots) on each trial. Red dots are spikes of the neuron. Third column: similar to the second column, shown for homing. **b**, Two trial matrices shown for the first grid cell example in **a** during search. The left trial matrix shows the firing rate as a function of the mouse’s position along the *y* axis of the arena (Y-position). The right matrix shows the neuron’s firing rate as a function of the distance between the mouse and the lever box. Firing rate as a function of Y-position or lever distance is shown on top of the trial matrix. **c**, Trial matrix of grid cell firing rate as a function of the mouse position along the *y* axis of the arena (Y-position, left) and distance between the mouse and the lever box (right) during homing, similar to **b**. **d**, Mean trial matrix correlation for all grid cells obtained from trial matrices with *y*-axis coordinate (top) or distance to the lever box (bottom). Data for search and homing behavior are shown separately (*N* = 17 mice, two-sided Wilcoxon sign-rank test, Y-position: Search Dark versus Homing Dark: *P* = 1.526 × 10^−5^; Lever distance: Search Dark versus Homing Dark: *P* = 7.629 × 10^−5^); Data are presented as mean values ± s.e.m. **e**, Trial matrix of grid cell firing rate as a function of different variables: Distance, Y-position, Lever distance and Time during search (top) and homing (bottom). Data for dark trials are shown (*N* = 17 mouse, two-sided Wilcoxon sign-rank test with Holm–Bonferroni correction, Search: Distance versus Y-position: *P* = 1.068 × 10^−3^, Y-position versus Time: *P* = 4.639 × 10^−3^; Homing: Lever distance versus Distance: *P* = 1.343 × 10^−3^, Lever distance versus Y-position: *P* = 1.526 × 10^−5^); Data are presented as mean values ± s.e.m. Source data

**Extended Data Fig. 6:**
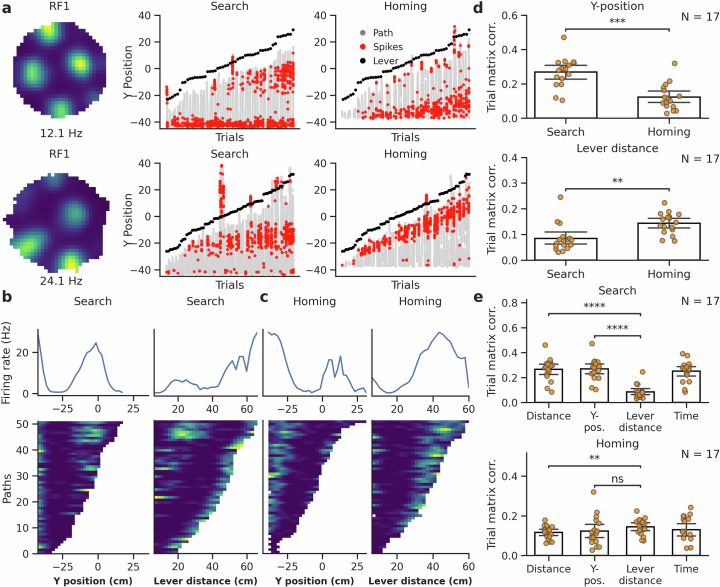
Firing fields of grid cells modulated by lever location in light. **a**, Examples of two grid cells in light. First column: Firing rate maps during random foraging. Second column: Firing of the neuron plotted as a function of mouse position on the y-axis of the arena (Y-position, 0 representing the arena’s center and −40 marking the edge near the bridge) during search. All search paths (gray lines) are shown next to each other and ranked according to the Y-position of the lever box (black dots) on each trial. Red dots are spikes of the neuron. Third column: similar to the second column, shown for homing. **b**, Two trial matrices shown for the first grid cell example in a during search. The left trial matrix shows the firing rate as a function of the mouse’s position along the y-axis of the arena (Y-position). The right matrix shows the neuron’s firing rate as a function of the distance between the mouse and the lever box. Firing rate as a function of y-position or lever distance is shown on top of the trial matrix. **c**, Trial matrix of grid cell firing rate as a function of mouse position along the y-axis of the arena (Y-position, left) and distance between the mouse and the lever box (right), similar to b. **d**, Mean trial matrix correlation for all grid cells obtained from trial matrices with y-axis coordinate (top) or distance to the lever box (bottom). Data for search and homing behavior are shown separately (N = 17 mouse, two-sided Wilcoxon sign-rank test, Y-position: Search Light vs. Homing Light: P = 1.526 ×10^−4^; Lever distance: Search Light vs. Homing Light: P = 2.090 ×10^−3^); Data are presented as mean values +/- SEM. **e**, Trial matrix of grid cell firing rate as a function of different variables: Distance, Y-position, Lever distance and Time during search (Top) and homing (Bottom). Data for light trials are shown (N = 17 mouse, two-sided Wilcoxon sign-rank test with Holm-Bonferroni correction, Search: Distance vs. Lever distance: P = 1.526 ×10^−5^, Y-position vs. Lever distance: P = 1.526 ×10^−5^; Homing: Lever-distance vs. Distance: P = 1.068 ×10^−3^, Lever-distance vs. Y-position: P = 1.202 ×10^−1^); Data are presented as mean values +/- SEM. Source data

Since grid cells indicated some degree of spatial coherence during the AutoPI task (Fig. 2g), we hypothesized that they might encode for positional information during the AutoPI task. We first characterized the spike-on-path of grid cells during search and homing of each trial. During search in darkness, we found that many grid cells showed firing fields at a constant position (Y-position) on the arena. In contrast, during homing in darkness, the position of a firing field was predominantly determined by the distance of the mouse from the lever (Fig. 3a). Interestingly, during homing in light trials, some grid cells showed firing fields at a constant Y-position on the arena, while others showed firing fields determined by distance of the mouse from the lever (Extended Data Fig. 6a). We constructed trial matrices^30^ for both the search and homing behavior of the task. Each trial matrix contains the firing rate of a single neuron across multiple trials, with each trial represented by a separate row. This arrangement allows the assessment of the consistency and reliability of firing patterns across trials. For trial matrices, firing rates were plotted against either the mouse’s Y-position on the arena or the distance between the mouse and the lever box (Fig. 3b,c and Extended Data Fig. 6b,c). During search in dark or light trials, trial matrix correlation for the Y-position was higher compared to homing, whereas correlation based on distance to the lever was larger during homing than search (Fig. 3d and Extended Data Fig. 6d).

To identify the variable with the highest trial matrix correlation, we compared grid cell firing correlations based on distance traveled, Y-position, distance to the lever and time. During search behavior in darkness, the trial matrix correlation for Y-position was significantly higher than those for distance traveled and time (Fig. 3e). In contrast, during homing behavior in darkness, the distance to the lever showed the highest trial matrix correlation (Fig. 3e). Interestingly, during homing tasks under light conditions, no significant difference was observed between the trial matrix correlations for Y-position and distance to the lever (Extended Data Fig. 6e). These results indicate that despite the absence of visible grid pattern, grid cells retain some degree of positional coding, which is influenced by the changing lever location on different trials.

### Preserved temporal firing associations and phase relationships during the path integration task

**Fig. 4:**
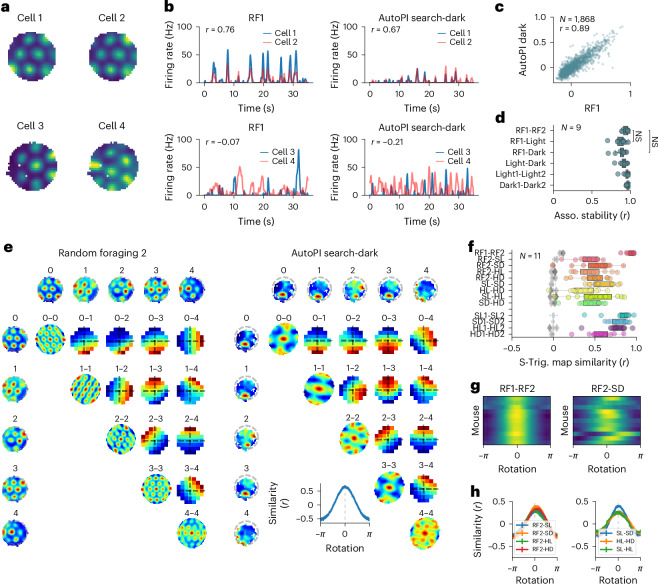
Preserved temporal relationships and spatial offsets of grid cells across behaviors. **a**, Firing rate maps of two pairs of grid cells recorded simultaneously. The grid cells on the top row had overlapping firing fields, whereas the ones on the bottom row had nonoverlapping firing fields. **b**, Instantaneous firing rate of the grid cell pairs shown in **a** during random foraging and the AutoPI task. The correlation coefficient between the firing rate of the two neurons is shown (*r* value). **c**, Firing associations of all grid cell pairs recorded on different probe shanks during the first random foraging trial and the AutoPI task (dark trials). Each data point is the correlation coefficient (*r* value) of a grid cell pair in the first random foraging trial and the AutoPI task (dark trials). Firing associations were strongly correlated across behavioral conditions (*N* = 1,863, two-sided Pearson *r* = 0.89, crossed fixed-effects model: fixed-effects estimate *e* = 0.81, *P* < 2.00 × 10^−16^). **d**, Correlation of firing association for pairs of grid cells across different behavioral conditions (one data point per mouse, 11 mice with at least 10 grid cell pairs, two-sided Wilcoxon signed-rank test, RF1-RF2 versus RF1-Dark: statistic = 6.0, *P* = 0.055, RF1-RF2 versus RF1-Light: statistic = 6.0, *P* = 0.055 RF1-RF2 versus Light-Dark: statistic = 12.0, *P* = 0.25). NS, not significant. Demonstrating preserved temporal associations of grid cell pairs across conditions. Box plots show the median (center line), first and third quartiles (box bounds) and 1.5 times the interquartile range (whiskers). **e**, Spike-triggered cross-firing rate map between pairs of grid cells in random foraging (left) and Search Dark (right). Five co-recorded grid cells are shown for these two conditions. The column of maps on the left and the upper row of maps correspond to the firing rate map of these grid cells in random foraging (left) or Search Dark (right). Autocorrelations are shown in the diagonal of the figure. The remaining maps are the spike-triggered cross-firing rate maps of each grid cell pair. Spike-triggered cross-firing rate maps are calculated by taking the spikes of grid cell A as reference and calculating the firing rate of grid cell B in close temporal relation. Map similarity of the two conditions are plotted after different rotations of one condition (lower-right figure). Error bars are indicated by vertical lines. **f**, Spike-triggered map similarity (*r* value) of grid cells across different conditions. Dots are the medians of individual mice. The shuffling distribution is shown in gray (11 mice with at least 10 grid cell pairs; RF1-RF2: median *r* value = 0.923, *P* = 2.117 × 10^−33^ (Tippett’s method), *P* = 9.766 × 10^−4^ (one-sample two-sided Wilcoxon signed-rank test); RF2-Search Light (SL): median *r* value = 0.430, *P* = 1.621 × 10^−19^ (Tippett’s method), *P* = 9.766 × 10^−4^ (one-sample two-sided Wilcoxon signed-rank test); RF2-Search Dark (SD): median *r* value = 0.520, *P* = 2.780 × 10^−20^ (Tippett’s method), *P* = 1.953 × 10^−3^ (one-sample two-sided Wilcoxon signed-rank test); RF2-Homing Light (HL): median *r* value = 0.432, *P* = 2.996 × 10^−19^ (Tippett’s method), *P* = 9.766 × 10^−4^ (one-sample two-sided Wilcoxon signed-rank test); RF2-Homing Dark (HD): median *r* value = 0.499, *P* = 3.012 × 10^−19^ (Tippett’s method), *P* = 9.766 × 10^−4^ (one-sample two-sided Wilcoxon signed-rank test); SL-SD: median *r* value = 0.545, *P* = 3.245 × 10^−25^ (Tippett’s method), *P* = 9.766 × 10^−4^ (one-sample two-sided Wilcoxon signed-rank test); HL-HD: median *r* value = 0.328, *P* = 1.340 × 10^−16^ (Tippett’s method), *P* = 1.953 × 10^−3^ (one-sample two-sided Wilcoxon signed-rank test); SL-HL: median *r* value = 0.502, *P* = 7.584 × 10^−21^ (Tippett’s method), *P* = 9.766 × 10^−4^ (one-sample two-sided Wilcoxon signed-rank test); SD-HD: median *r* value = 0.539, *P* = 1.498 × 10^−22^ (Tippett’s method), *P* = 9.766 × 10^−4^ (one-sample two-sided Wilcoxon signed-rank test); SL1-SL2: median *r* value = 0.857, *P* = 2.405 × 10^−26^ (Tippett’s method), *P* = 9.766 × 10^−4^ (one-sample two-sided Wilcoxon signed-rank test); SD1-SD2: median *r* value = 0.771, *P* = 2.314 × 10^−29^ (Tippett’s method), *P* = 9.766 × 10^−4^ (one-sample two-sided Wilcoxon signed-rank test); HL1-HL2: median *r* value = 0.755, *P* = 4.767 × 10^−22^ (Tippett’s method), *P* = 9.766 × 10^−4^ (one-sample two-sided Wilcoxon signed-rank test); HD1-HD2: median *r* value = 0.576, *P* = 1.098 × 10^−23^ (Tippett’s method), *P* = 9.766 × 10^−4^ (one-sample two-sided Wilcoxon signed-rank test). Demonstrating largely preserved spatial offset of grid cell pairs. Box plots show the median (center line), first and third quartiles (box bounds) and 1.5 times the interquartile range (whiskers). **g**, Heat map of map similarity between random foraging 1 (RF1) and random foraging 2 (RF2) (left), and between RF2 and SD conditions (right), for different rotations of one condition. Each row represents data from a single mouse. **h**, Rotation–correlation curves of map similarity between different conditions in the task. The similarity is assessed by rotating one condition and plotting the correlation with the other condition. Error bars are indicated by vertical lines. Source data

Previous work suggests that grid cell activity spans a toroidal manifold that does not change across behavior or brain states^33–35^. If this is also the case during the path integration task, the temporal firing associations and the phase relationships between grid cells observed during random foraging and during the AutoPI task should be similar. The firing association between two grid cells was defined as the Pearson correlation coefficient between the instantaneous firing rate of the two cells. Figure 4a,b shows the firing associations of two grid cell pairs. The grid cell pairs had either high or low temporal correlation of their instantaneous firing rates during the first random foraging trial, respectively. These firing associations were preserved during the AutoPI task. When considering all grid cell pairs from sessions with at least ten pairs (from 11 mice), the correlation between their firing association during random foraging and during dark trials was 0.89 (Fig. 4c and Supplementary Fig. 1). The correlation of the firing association between the first and the second random foraging trial compared to the first random foraging trial and the light or dark trials of the AutoPI task was close to 1 in all cases and thus not significantly different (Fig. 4d). This demonstrates that the temporal associations of grid cell pairs were preserved across all our recording conditions.

We assessed the relative spatial offset of grid cells over short time intervals (5 s) using spike-triggered cross-firing rate maps in sessions with at least ten grid cell pairs (Fig. 4e). We found that these maps were significantly correlated across behavioral conditions (random foraging and different phases of the task), albeit less than within most conditions (Fig. 4f), suggesting that spatial offset of grid cell pairs are largely maintained. We then rotated the spike-triggered cross-firing maps in one condition by different amounts and assessed map similarity with the other condition. The peak map similarity was observed with no systematic rotation (0°) in most mice (Fig. 4g), showing that the orientation of the grid cells was most often preserved across conditions (Fig. 4g,h). Overall, despite the absence of grid patterns during the AutoPI task, the temporal association as well as spatial offset of grid cell pairs are largely maintained, suggesting that the internal manifold of grid cell population dynamics is preserved.

### Moment-to-moment decoding of movement path from grid cell activity

**Fig. 5:**
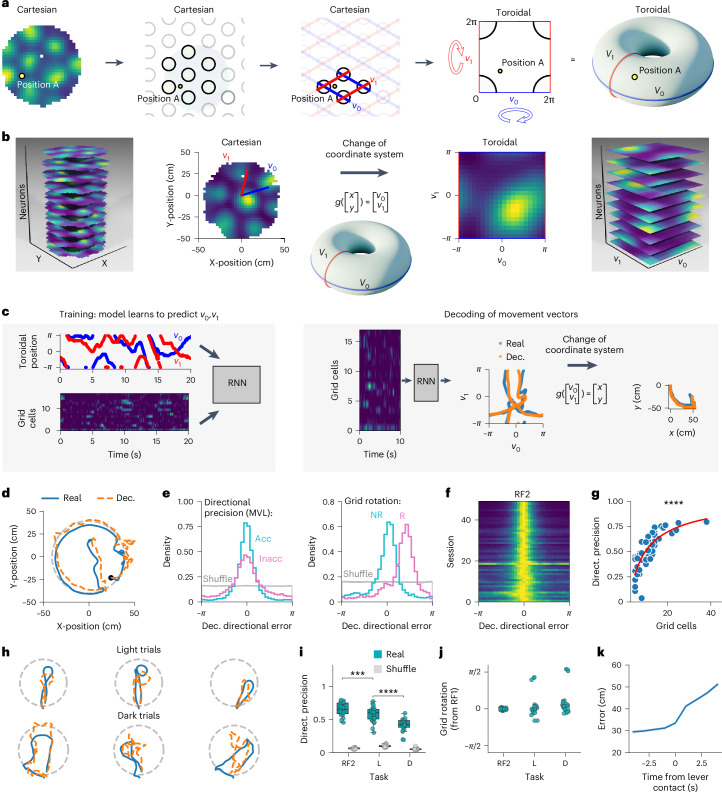
Decoding of the animal’s movement vectors from grid cell activity. **a**, Schematic explaining the transformation of Cartesian position data to a toroidal coordinate system. A detailed explanation is presented in Supplementary Video 1. **b**, Transforming grid cells in Cartesian space onto the toroidal manifold. Left, Stack of grid cell firing rate maps from random foraging. Grid orientation and period were estimated to define the two main grid axes (*v*_0_ and *v*_1_). Right, Firing rate maps of the same grid cells in toroidal space. **c**, Left, A RNN was trained to decode the animal’s position in toroidal space (*v*_0_ (blue) and *v*_1_ (red)) from the instantaneous firing rate of simultaneously recorded grid cells. The data from the first random foraging trial were used for training. Right, The trained RNN decoded the animal’s position in toroidal space. Toroidal movement vectors were calculated from the change in toroidal position over time. The toroidal vectors were transformed into Cartesian vectors and were summed to reconstruct movement paths. **d**, Example of real (blue) and decoded (orange) paths during the second random foraging trial. 25 s are shown. The light-gray dashed line represents the arena border. Movement vectors decoded by the model were added to the real position of the mouse at the start of the decoding period for reconstruction. **e**, Two measures derived from the distribution of decoded directional error. The data from two sessions are shown (light blue and pink). The MVL of these distributions reflects the directional precision of the model (left), whereas their circular means represent the rotation of the grid representation relative to the first random foraging trial (right). The data from two sessions are shown (light blue representing a session with high MVL and low rotation and pink representing an example session with low MVL and significant rotation. Acc, accurate directional precision; Inacc, inaccurate directional precision; NR, no rotation; R, rotation). Shuffled distribution is shown in gray. **f**, Distribution of decoded directional error for 49 recording sessions with at least five grid cells during the second random foraging trial (RF2). **g**, Directional precision as a function of the number of grid cells in the model (Pearson correlation, two-sided, *N* = 49 sessions, *r* = 0.736, *P* = 1.67 × 10^−9^). The data were fitted with a logarithmic equation of the second degree (red curve). **h**, Examples of real (blue) and decoded (orange) paths during the AutoPI task. **i**, Directional precision during RF2 and light (L) and dark (D) trials on the AutoPI task compared to shuffled data (*N* = 24 sessions from seven mice, two-sided Wilcoxon signed-rank test, RF2-L: statistic = 35.0, *P* = 0.00049, L-D: statistic = 3.0, *P* = 5.96 × 10^−7^). Box plots show the median (center line), first and third quartiles (box bounds) and 1.5 times the interquartile range (whiskers). **j**, Rotation of the grid representation during RF2 and L and D trials on the AutoPI task. **k**, Cumulative error in position decoding as a function of time from finding the lever during dark trials. ****P < 0.0001, ***P < 0.001. Source data

To directly test if the grid cell network reflected path integration during the homing task despite the lack of grid pattern in traditional firing rate maps, we developed a computational framework to decode the mouse’s ongoing movement vectors based on grid cell activity (Fig. 5). The rationale behind this decoding model and a step-by-step guide through the computational procedure are provided in Supplementary Video 1. We took advantage of the fact that the activity of grid cells spans the surface of a torus and that the activity manifold is preserved across behaviors^32,34^. We only used sessions with at least five simultaneously recorded grid cells from the same module (that is, with similar orientation and spacing; 49 sessions from 11 mice). The first random foraging trial was used to establish the orientation and period of the grid pattern and transform the Cartesian position data (*x*, *y*) into coordinates in toroidal space (*v*_0_, *v*_1_; Fig. 5a,b). We trained a recurrent neural network (RNN) to decode the animal’s position in toroidal space based on the instantaneous firing rate of the grid cells (Fig. 5c). To decode the movement vectors of the mouse, we calculated the change in the decoded toroidal position over time, resulting in a series of movement vectors in toroidal space. These movement vectors were then transformed from toroidal space to Cartesian space. The cumulative sum of the Cartesian movement vectors represented the decoded movement path (Fig. 5c and Supplementary Video 2).

Figure 5d shows the similarity between real and decoded paths during random foraging. To quantify the quality of the movement decoding, we calculated the angle between the real and decoded movement directions, referred to as decoded directional error. From the distribution of decoded directional error, we extracted two principal measurements: directional precision and grid rotation (Fig. 5e). The directional precision was defined as the mean vector length (MVL) of the decoded directional error distribution, whereas the grid rotation relative to the first random foraging trial was obtained by calculating the circular mean of the decoded directional error distribution. There was no systematic rotation of the grid pattern between the first and second random foraging trials (Fig. 5f; 359.65° ± 2.26°, *N* = 24 sessions, Wilcoxon signed-rank test: statistic = 125.0, *P* = 0.491). The directional precision of the model increased with the number of grid cells participating in the decoding (Fig. 5g). For recording sessions with at least 15 grid cells (*N* = 9), the directional precision when running speed exceeded 10 cm s^−1^ had a median of 0.76 (MVL), a degree of directional selectivity comparable to sharply tuned head-direction cells^36–39^. Additionally, analysis using simulated grid cells revealed that model accuracy improves when the grid spacing is smaller and when the grid cells are more widely dispersed across the toroidal manifold (Supplementary Fig. 2).

**Extended Data Fig. 7:**
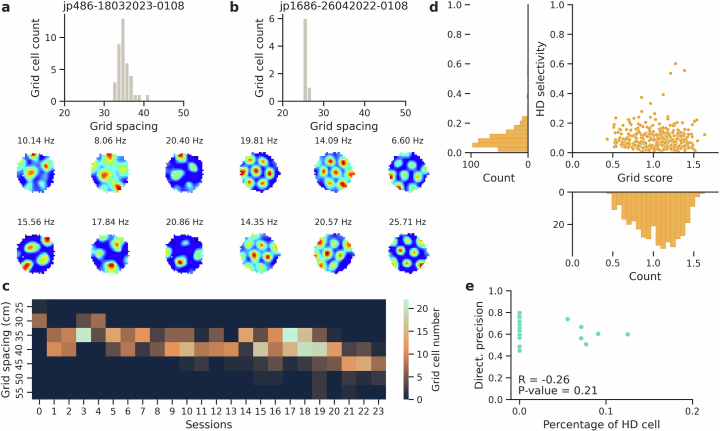
Grid spacing and head-direction selectivity of grid cells used for reconstruction. **a**, Top: Distribution of grid spacing of recorded grid cells from one session. Bottom: Examples of 2D firing map from six grid cells recorded in this session. **b**, Similar to A, but showing a session with a different grid module. **c**, Distribution of grid spacing of simultaneously recorded grid cells from sessions used for decoding grid cell activities (n = 24 sessions from 7 mice; These were sessions with a directional precision larger than 0.5 in the second random foraging out of 49 sessions from 11 mice). **d**, Grid score and head direction selectivity of grid cells used for the model reconstruction. **e**, Pearson correlation between the percentage of conjunctive grid-head direction cells and directional precision decoding of the model (r = −0.26, *P* = 0.21). Source data

**Extended Data Fig. 8:**
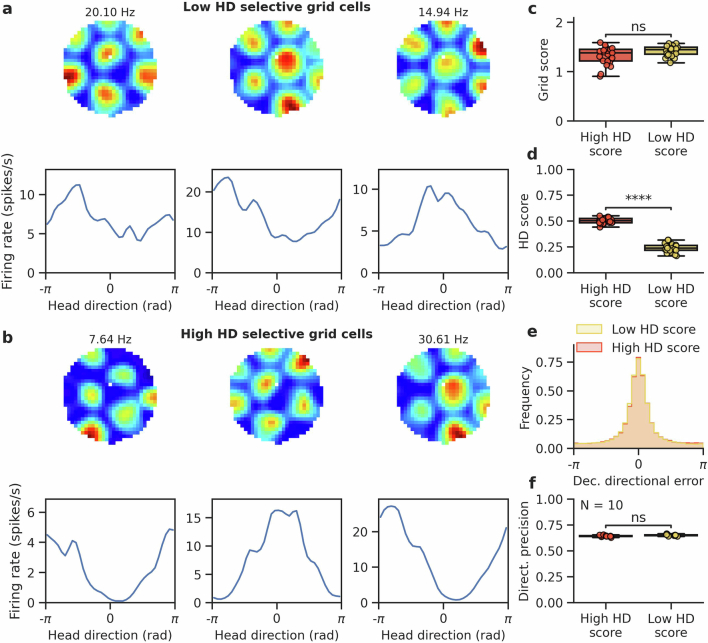
Head-direction selectivity has no effect on model performance. **a-b**, To test the effect of head direction (HD) selectivity on model performance, we simulated groups of grid cells where they differ in HD selectivity. Three examples are shown for each group (**a**: Low HD selectivity; **b**: High HD selectivity) of 25 simulated grid cells. First row: firing rate map of grid cell examples, Second row: Head-direction selectivity of grid cell examples. **c**, Grid score between high HD selectivity grid cell group and low HD selectivity grid cell group (N = 25 grid cells, Mann-Whitney-Wilcoxon test two-sided: *P* = 5.003 ×10^−2^). Box plots show median (center line), first and third quartiles (box bounds), and 1.5 × interquartile range (whiskers). **d**, HD score between high HD selectivity grid cell group and low HD selectivity grid cell group (N = 25 grid cells, Mann-Whitney-Wilcoxon test two-sided: *P* = 1.416 ×10^−9^). Box plots show median (center line), first and third quartiles (box bounds), and 1.5 × interquartile range (whiskers). **e**, Distribution of decoded directional error of model decoding trained on low HD score grid cell group and high HD score grid cell group. **f**, Simulation for low and high HD selectivity was performed 10 times, comparisons are made between the directional precision of the model trained in the two groups (N = 10 independent test runs, Mann-Whitney-Wilcoxon test two-sided: *P* = 5.327 ×10^−2^). Box plots show median (center line), first and third quartiles (box bounds), and 1.5 × interquartile range (whiskers). ****P < 0.0001. Source data

We then applied the path reconstruction analysis to the AutoPI task for sessions where the directional precision during the second random foraging trial was above 0.5 (24 sessions from 7 mice; Supplementary Table 1 and Fig. 5h). For most mice (6 of 7), we recorded only one grid module (Extended Data Fig. 7a–c). The modulation of firing rate by head direction did not contribute to the directional precision of the model (Extended Data Figs. 7d,e and 8). Because half of the sessions came from one animal, we repeated the analysis excluding this mouse and showed that all major conclusions were maintained (Supplementary Fig. 11). Directional precision decreased from random foraging to light trials of the AutoPI task and from light trials of the AutoPI task to dark trials (Fig. 5i). The directional precision during dark trials of the AutoPI task was higher than chance levels for all sessions in which path reconstruction was attempted (24 of 24 sessions), demonstrating that self-motion-based cues are sufficient for grid cell modules to encode approximate movement vectors. The orientation of the grid representation was maintained from random foraging to the AutoPI task in all but one mouse (Fig. 5j). During dark trials of the AutoPI task, the error accumulation rate in the decoded integrated path increased once the mouse reached the lever (Fig. 5k).

### Reanchoring of grid cells during navigation in darkness

**Fig. 6:**
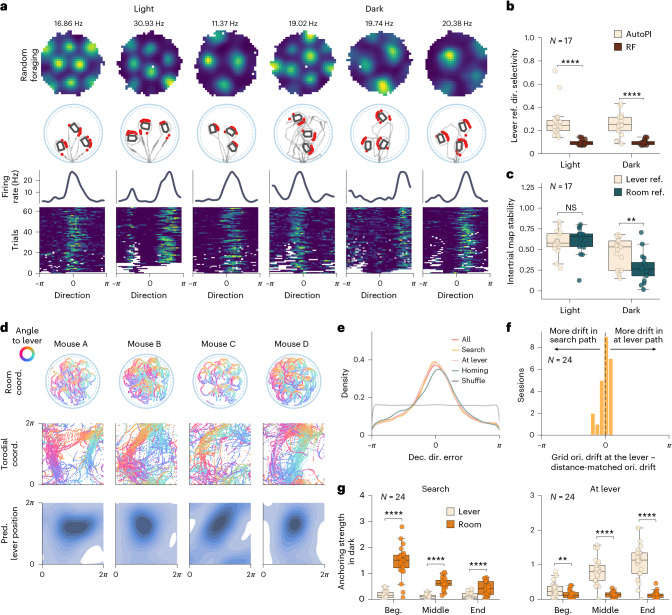
Grid cell modules reanchor to the lever location. **a**, Examples of six grid cells with firing fields near the lever location in light and dark trials. First row, Firing rate maps during random foraging. Second row, Spikes (red) on the running path (gray) during three trials. Only spikes emitted within 18 cm of the lever are shown. Third row, Firing rate as a function of the direction of the animal relative to the lever center. Fourth row, Firing rate as a function of direction around the lever on different trials. Each row represents a trial in which the mouse pressed the lever. **b**, Directional selectivity (MVL) of neurons in the lever-centered reference frame in light and dark trials in the AutoPI task against a null condition generated with random foraging in the same session. The null condition is generated by using the lever positions from light or dark trials of the AutoPI task and applying them to the random foraging data. We ensured that the occupancy at each lever position (18 cm around the lever) during random foraging matches the actual occupancy observed during the task. From this, we generated lever reference frame firing rate maps separately for light and dark trials. The directional selectivity of these lever-centered maps was then calculated and used as the null condition (*N* = 17 mice with grid cells with lever-anchored fields, two-sided Wilcoxon sign-rank test, Light: *P* = 1.526 × 10^−5^, Dark: *P* = 7.629 × 10^−5^). Box plots show the median (center line), first and third quartiles (box bounds) and 1.5 times the interquartile range (whiskers). **c**, Intertrial stability of grid cells with lever-anchored firing fields. Intertrial stability was defined as the Pearson correlation coefficient between the firing rate maps generated from two stacks of randomly assigned trials of the specified light condition. Maps were calculated in the room reference frame or the lever reference frame (*N* = 17 mice with grid cells with lever-anchored fields, two-sided Wilcoxon sign-rank test, light: *P* = 6.777 × 10^−1^, dark: *P* = 2.090 × 10^−3^). Box plots show the median (center line), first and third quartiles (box bounds) and 1.5 times the interquartile range (whiskers). **d**, Anchoring of grid cell modules to the lever position during dark trials. Top, Examples of the running paths of the mouse around the lever during recording sessions from four different mice (mouse running speed > 10 cm s^−1^). The mouse’s path is color coded to reflect the direction of the mouse around the lever. Middle, Decoded position of the mouse in toroidal space when the mouse is around the lever. The color code represents the direction of the mouse relative to the lever. Bottom, Kernel density estimate of the decoded lever position in toroidal space when the mouse is near the lever. **e**, Distribution of decoded directional error for all dark trials or during three separate phases (search, at lever and homing) of the dark trials. **f**, Distribution of the difference between grid orientation drift at the lever and distance-matched orientation drift for each trial, averaged across the 24 sessions (*N* = 24 sessions, one-sample Wilcoxon signed-rank test, *P* = 0.375). For further details, see Supplementary Fig. 6c. **g**, Anchoring strength to the lever or the room reference frame during dark trials. Data during the search behavior and when the mouse was at the lever are shown separately and split evenly into the beginning, middle and end (*N* = 24 sessions, two-sided Wilcoxon sign-rank test, Search, Beg.: *P* = 1.192 × 10^−7^, Middle: *P* = 2.384 × 10^−7^, End: *P* = 9.084 × 10^−5^; At lever, Beg.: *P* = 8.905 × 10^−3^, Middle: *P* = 2.384 × 10^−7^, End: *P* = 3.576 × 10^−7^). Box plots show the median (center line), first and third quartiles (box bounds) and 1.5 times the interquartile range (whiskers). ****P < 0.0001, ***P < 0.001, *0.01 < *P* < 0.05; NS, 0.05 < *P*. Source data

**Extended Data Fig. 9:**
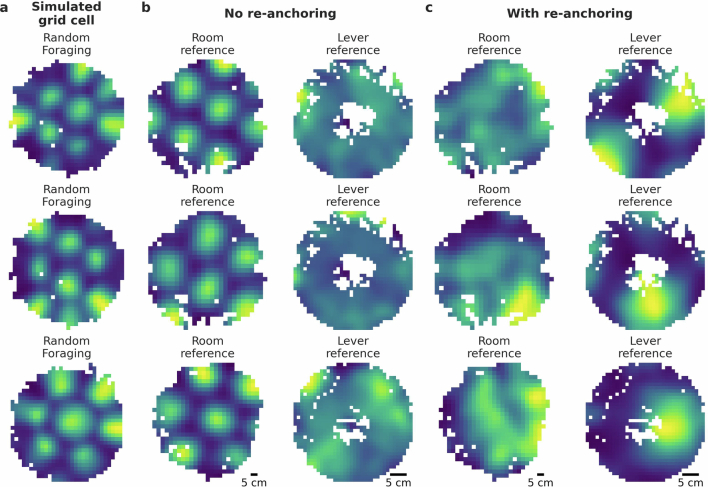
Firing rate map of simulated grid cells with and without re-anchoring to the lever. **a**, Firing rate maps of three example grid cells in the first random foraging. **b**, Firing rate map of simulated grid cells without re-anchoring to the lever during the task using the real position of the animal. Simulated firing rate maps are shown both in the room reference frame (left, bin size = 3 cm) and lever reference frame (right, bin size = 1 cm). **c**, Firing rate map of simulated grid cells with re-anchoring to the lever during each trial. Similar to b, simulations are shown for the room (left) and lever reference (right). Source data

**Extended Data Fig. 10:**
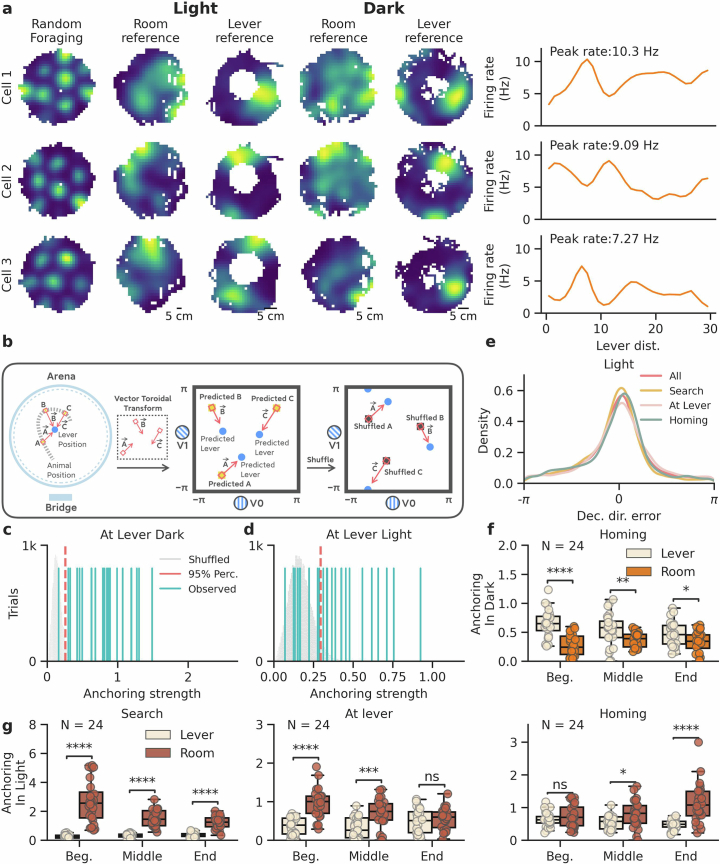
Re-anchoring of grid cell modules to the lever location. **a**, Examples of three grid cells with firing fields near the lever location. Firing rate map of light and dark trials in the arena reference frame and lever reference frame are shown. Firing rate as a function of distance from the lever are also shown. **b**, Graphical abstract of anchoring strength assessment. To measure this, we compute the vector from the animal’s position to the lever and transform this vector into the toroidal space. This vector is added to the decoded animal position as predicted by the model, yielding the predicted lever position for each decoded point. This analysis tests whether lever positions cluster in toroidal space to determine grid module anchoring. If anchored to the room reference frame, decoded positions vary with different lever location; if anchored to the lever frame, they would cluster. By decoding over time, we can assess the anchoring strength to the lever throughout the trial. **c**, Strength of the grid anchoring to the lever in dark trials. The gray distribution is the anchoring strength expected by chance (shuffling 1000 times). The red line denotes the 95th percentile. Out of 24 sessions, 23 show an above-chance anchoring to the lever. **d**, Strength of the grid anchoring to the lever in light trials. The gray distribution is the anchoring strength expected by chance (shuffling 1000 times). The red line denotes the 95th percentile. Out of 24 sessions, 15 show an above-chance anchoring to the lever. **e**, Probability density map of the angle of rotation between the movement vector decoding of the model against the actual movement vector of the mouse (Decoded directional error). Data is shown for all light trials in four different conditions in the task. **f**, Anchoring strength to the lever or the room reference during homing in dark trials. The intervals for homing are split evenly into the beginning, middle, and end. (N = 24 sessions, two-sided Wilcoxon sign-rank test, Homing, Beg.: *P* = 8.345 ×10^−6^, Middle: *P* = 4.335 ×10^−3^, End: *P* = 1.260 ×10^−2^). Box plots show median (center line), first and third quartiles (box bounds), and 1.5 × interquartile range (whiskers). **g**, Anchoring strength to the lever or the room reference in light trials. The intervals for search (left), at lever (middle), and homing (right) are plotted separately and split evenly into the beginning, middle, and end. Data is shown for Search, At Lever, and Homing conditions during the task (N = 24 sessions, two-sided Wilcoxon sign-rank test, Search, Beg.: *P* = 1.192 ×10^−7^, Middle: *P* = 1.192 ×10^−7^, End: *P* = 1.192 ×10^−7^; At lever, Beg.: *P* = 1.192 ×10^−6^, Middle: *P* = 4.299 ×10^−4^, End: *P* = 5.271 ×10^−1^; Homing, Beg.: *P* = 2.292×10^−1^, Middle: *P* = 1.378 ×10^−2^, End: *P* = 8.345 ×10^−6^). Box plots show median (center line), first and third quartiles (box bounds), and 1.5 × interquartile range (whiskers). ****P < 0.0001, ***P < 0.001, ** 0.001 < *P* < 0.01, * 0.01 < *P* < 0.05, ns 0.05 < *P*. Source data

During the AutoPI task, we observed decreased path prediction accuracy after the mouse reached the lever (Fig. 5j) and modulation of the grid cell pattern by the lever location during homing (Fig. 3). We hypothesized that this could be caused by a reanchoring of the grid pattern to the randomly located lever. We first characterized the firing fields of grid cells when the mouse ran around the lever. We found that many grid cells fired when the mouse was located in a specific direction relative to the center of the lever (Fig. 6a). Importantly, these fields were present in most trials even though the position of the lever changed across trials. Directional selectivity of these fields corresponds to a vector from the lever to the mouse position when the grid cell fires. The lever-referenced directional selectivity of grid cells around the lever during light or dark trials was significantly higher than chance levels (Fig. 6b). These lever-anchored grid fields suggest that grid cells could be influenced by two reference frames on the AutoPI task: a room reference frame and a lever-centered reference frame (Extended Data Fig. 9). To assess their respective impact, we calculated firing rate maps in both reference frames for periods when the mouse was close to the lever (Extended Data Fig. 10a). During dark trials, the intertrial stability of firing rate maps was higher in the lever-centered reference frame than in the arena reference frame (Fig. 6c). To evaluate the internal grid cell manifold following a change in reference frames, we analyzed spike-triggered cross-firing rate maps when the animal was at the lever (Supplementary Fig. 3a–c). We found a significant correlation between the maps during random foraging and when the animal was at the lever, with the highest map similarity observed without any rotation (0°) in the majority of mice (Supplementary Fig. 3d,e). This preservation of spatial offsets in grid cell pairs suggests that the internal grid cell manifold remains largely intact when the mouse is at the lever (after reanchoring). Interestingly, a subset of non-grid cells with fields around the lever were engaged specifically during task-relevant interactions with the lever, potentially contributing to the reanchoring of grid cells based on behavioral context (Supplementary Fig. 4).

We tested the hypothesis that grid cell modules anchor to the lever position using the RNN decoding the animal position in toroidal space (Fig. 5b). At the grid module level, anchoring to an object implies that this object has a stable location in toroidal space, irrespective of the location of the object on the arena. Figure 6d shows the animal’s position as it ran around the lever in Cartesian space and the decoded position in toroidal space (Fig. 6d). In several recording sessions, the decoded paths in toroidal space when the animal ran around the lever formed a ring (Supplementary Fig. 5). This ring was particularly evident during dark trials. To estimate the position of the lever in toroidal space, we obtained the Cartesian vector from the animal’s position to the lever and added the equivalent toroidal vector to the decoded toroidal position of the mouse (Extended Data Fig. 10b). When the mouse was near the lever, the lever position in toroidal space clustered at a specific location (Fig. 6d). We fitted a bivariate von Mises distribution to the lever positions in toroidal space and used the two kappa parameters to measure grid anchoring strength. During dark trials, the grid anchoring to the lever was statistically significant in 23 of 24 sessions (Extended Data Fig. 10c, for comparison, see Extended Data Fig. 10d). The decoded directional error distribution was centered around zero, suggesting that the grid representation is centered around a baseline within each condition (Fig. 6e and Extended Data Fig. 10e). Furthermore, trial-by-trial orientation comparisons revealed that encountering the lever did not produce greater orientation drift than during a distance-matched search path (Fig. 6f and Supplementary Fig. 6a,c–f), indicating that there was no systematic rotation of the grid pattern when the mouse was around the lever. This demonstrates that the reanchoring to the lever occurred via a translation of the grid pattern. To determine if the phase change during reanchoring is coherent across all recorded grid cells or involves firing location changes for individual neurons, we simulated these scenarios and compared them to our observations (Supplementary Fig. 7). Our results are compatible with a single or multiple coherent phase change(s) for grid cells within the same module.

Using the same method to assess grid anchoring strength, we assessed how grid anchoring evolved during single runs on the arena (Fig. 6g). Grid modules were firmly anchored to the room reference frame at the beginning of the search path, but the dominant anchoring transitioned to the lever position after the mouse reached the lever. After departing from the lever, the degree of anchoring to the lever decreased, particularly in the later stages of the homing behavior (Extended Data Fig. 10f). These conclusions were maintained when correcting for trial-by-trial rotations of the grid axis (Supplementary Fig. 8). Thus, the observed steep increase in decoding error at the time of lever contact in dark trials is most likely due to the anchoring of the grid module to the lever (Supplementary Figs. 6b and 9), a landmark without a fixed position in Cartesian space. Interestingly, during homing in light trials, grid modules increasingly anchor back to the room reference before reaching the periphery (Extended Data Fig. 10g). This reanchoring, most likely using visual input, may explain the similar trial matrix correlations based on Y-position and lever distance observed in single grid cells during homing in light trials (Extended Data Fig. 6e).

### Grid orientation drift increases with running path length

**Fig. 7:**
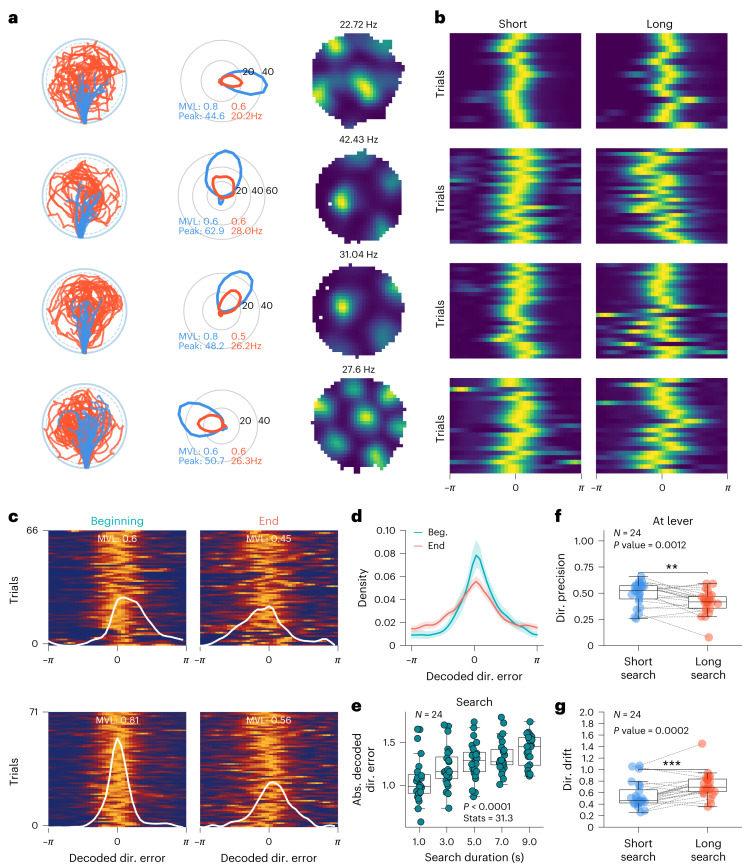
Increased variance in grid cell orientation following long search paths in darkness. **a**,**b**, Examples of four lever-anchored grid cells in dark trials from different sessions. Trials are split by the median of the search path into a short search path (blue) and a long search path (red). **a**, First column shows the short (blue) and long (red) search paths of the animal in dark trials. Second column shows a firing rate histogram of lever-anchored grid cell as a function of the direction of the mouse from the lever center. The MVL and peak firing rate during the short search path (blue) and the long search path (red) are shown. Third column shows firing rate maps of the four example grid cells during random foraging. **b**, Trial matrix representing the neuron’s firing rate as a function of direction to the lever center for both short and long search paths. Each row in the matrix represents the firing rate of the neuron in one trial, adjusted by the average angle of trial matrix direction to the center. The further the peak firing rate is from the center, the greater the deviation in grid cell orientation. **c**, Trial matrices of the decoded directional error during long search paths’ beginning (left) and end (right) of dark trials. Two recording sessions are shown, one per row. The white line shows the mean decoded directional error across trials. The MVL of the distribution is shown. The smaller the decoded directional error per trial, the higher the MVL, **d**, Probability density of the decoded directional error during the beginning and end of long search paths (*N* = 24 sessions from seven mice, two-sided Kolmogorov–Smirnov Test, statistic = 0.264, *P* = 7.492 × 10^−27^); Data are presented as mean values ± s.e.m. **e**, Absolute decoded directional error as a function of search duration during dark trials (*N* = 24 sessions from seven mice, two-sided Friedman test, statistic = 31.30, *P* = 7.349 × 10^−7^). **f**, Directional precision when the mouse was at the lever after short or long search paths (*N* = 24 sessions from seven mice, two-sided Wilcoxon sign-rank test, *P* = 1.238 × 10^−3^). **g**, Directional drift when the mouse was at the lever after short and long search paths (*N* = 24 sessions, two-sided Wilcoxon sign-rank test, *P* = 2.393 × 10^−4^). Box plots show the median (center line), first and third quartiles (box bounds) and 1.5 times the interquartile range (whiskers). ***P < 0.001, **0.001 < *P* < 0.01. Source data

Although we found that the mean orientation of the grid pattern did not change from random foraging to the AutoPI task (Figs. 5i and 6e), we hypothesized that, in individual trials in darkness, the orientation of the grid pattern drifted from its original orientation. We first split the search path into long and short search paths (Fig. 7a) and examined the firing rate and directional selectivity of the grid fields when the animal arrived at the lever. During the short search paths, we observed higher peak firing rates (Fig. 7a) and greater MVLs (Fig. 7b) in the directional firing rate histograms of these cells. To test the directional drift in population grid cell activity as the animal searched for the lever in darkness, we compared the decoded directional error of the model (as in Fig. 5d) during the beginning and end of the search paths of dark trials (Fig. 7c). The directional precision of the model was higher at the beginning of the search compared to the end (Fig. 7c,d), suggesting that the orientation of the grid pattern drifts during the search path. We also observed a progressive increase in the absolute decoded directional error as a function of search duration (Fig. 7e). The directional precision was lower after long search paths than shorter ones during search (Supplementary Fig. 10) and when the mouse was at the lever (Fig. 7f). We also quantified the directional drift on single trials as the animal ran around the lever in darkness and found that the directional drift was higher after long than short search paths. (Fig. 7g). These results are consistent with the idea that grid cell orientation during dark trials is updated by an angular path integrator that accumulates error over time or distance run. Importantly, despite reanchoring events, the accumulated error by the path integrator during the search is preserved after the change of reference frames.

### Grid orientation drift predicts homing behavior

**Fig. 8:**
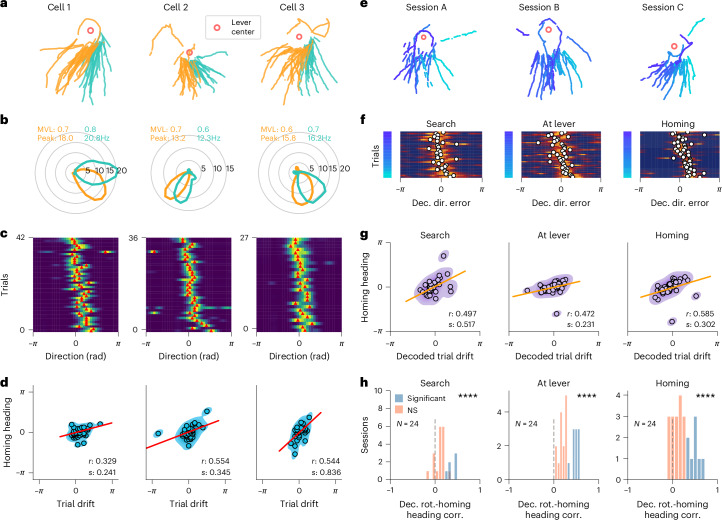
Grid cell orientation predicts homing behavior during navigation in darkness. **a**–**d**, Examples of three grid cells with lever-anchored firing fields that predicted homing direction. **a**, Trials were divided based on whether the mouse headed left (orange) or right (green) relative to the median homing direction of the session. Lever-centered homing paths of the animal in three sessions are shown. The red circle indicates the center of the lever. **b**, Firing rate of three grid cells as a function of the direction of the mouse relative to the lever when the mouse was near the lever. The data are plotted separately for trials in which the animal homed left or right relative to the median homing direction. The MVL and peak rate are indicated. **c**, Trial matrix showing the firing rate of the three grid cells as a function of the direction of the mouse relative to the lever. Each row represents one dark trial. The rows were sorted based on the homing direction of the mouse (from right to left). Red ticks indicate the peak firing direction for each trial. A peak firing rate shifted in a positive direction means that the field is shifted anticlockwise compared to the median. **d**, Trial drift of the neuron against the homing direction of the mouse. Points represent individual dark trials. A regression line (red), the circular correlation coefficient (*r*) and the slope of the regression line (*s*) are shown. **e**–**g**, Examples of correlation between the decoded directional error and homing direction. **e**, Lever-centered homing paths of the animal in three sessions. Paths are color coded by the mouse’s median heading direction while homing (homing heading). The red circle indicates the center of the lever. **f**, Trial matrix of the decoded directional error on single trials during the search (left), when the mouse was at the lever (middle) or during homing (right). The trials were sorted by the homing heading of the mouse, as indicated by the color code on the left of each matrix. A white circle indicates the circular mean in the decoded directional error distribution (decoded trial drift). When the decoded directional error is shifted in a positive direction, this means that the decoded path deviates from the real path in an anticlockwise direction. **g**, Correlation of decoded trial drift and homing heading error. Data points represent individual dark trials. The decoded trial drift was calculated when the mouse was at the lever (middle) or during homing (right). A regression line (orange), the circular correlation coefficient (*r*) and the slope (s) are shown. **h**, Distribution of circular correlation coefficients between the decoded directional error and homing heading. Significant and nonsignificant values are represented in blue and orange, respectively; *r* values were considered significant when exceeding the 97.5th percentile. Distributions are shown during the search path (top, *N* = 24 sessions, two-sided one-sample Wilcoxon signed-rank test, H_0_: median equals 0, statistic = 19.0, *P* = 3.659 × 10^−5^), when the mouse was at the lever (middle, statistic = 0.0, *P* = 1.192 × 10^−7^) and during the homing path (bottom, statistic = 20.0, *P* = 4.422 × 10^−5^). ****P < 0.0001, ***P < 0.001, ** 0.001 < *P* < 0.01. Source data

We investigated whether the grid orientation drift that accumulated during the search and as the animal ran near the lever predicted the direction of the mouse’s homing behavior. We divided the homing path of the animal into left and right homing trials (Fig. 8a), and found that the lever-anchored grid fields also drifted accordingly to the left and right homing direction of the animal (Fig. 8b). Additionally, we calculated the drift of the grid fields at the lever on each trial, and we found a significant correlation between the heading direction of the mouse and the grid cell firing field orientation around the lever (Fig. 8c,d). To understand how the drift in grid cells accumulated over time, we calculated the decoded directional error of the model separately during the search, while the mouse was at the lever and during homing (Fig. 8e,f). On each dark trial, we identified the circular mean in the decoded directional error distribution, which we refer to as decoded trial drift. We found that the decoded trial drift was correlated with the homing heading direction (Fig. 8f). Notably, the decoded trial drift of grid modules during the search, when the animal was around the lever, and during homing behavior was positively correlated with the animal’s homing heading direction (Fig. 8g). The distribution of *r* values for the decoded trial drift/homing direction correlations was significantly shifted toward positive values (Fig. 8h), demonstrating that the decoded trial drift of the grid map during the search path, at the lever, or during the homing path all predicted homing direction.

## Discussion

Grid cells have long been proposed as key components in the neural circuitry for path integration^3^. Previous models have proposed that these cells enable position coding from multiple grid scales^40^ or by encoding translation vectors that map start to goal locations through their firing patterns^24,27^. These models require a rigid organization of grid cells with minimal distortions to ensure consistent distance measurement across environments^41^. Furthermore, the grid map would need to be stable, so that positions and environmental landmarks are accurately represented within a single coordinate frame over extended periods^3,21^. Contrary to these expectations, our findings demonstrate that grid cells do not maintain a stable grid representation during a path integration task. Instead, we found a transition from a room reference frame to a local reference frame centered on the location of the lever. This reanchoring of the grid map involved a translation of the grid pattern with no change in grid orientation. Interestingly, a selective grid phase change without changes in grid orientation has previously been reported after changing nonmetric cues^42^ or the shape of an environment^43^, indicating that the grid phase can adapt to different local reference frames, while grid orientation remains constant in behavioral paradigms lasting over 10 min. Here, we demonstrate that a translational reanchoring of grid cell representation occurs within seconds when encountering a known landmark after a short exploratory journey in which position is estimated from self-motion cues. This anchoring of the grid maps to the lever could explain why some hippocampal place cells, which are located one or a few synapses downstream from grid cells^44^, have lever-anchored firing fields during this same behavioral task^30^. We predict that grid cells change their anchoring point in many navigational tasks where place cells have been shown to change reference frames^45–47^.

Grid map distortions in 2D spaces in irregularly shaped environments^48^, compartments^49^ or in three-dimensional environments^50^, as well as the shifting of single grid fields toward the home base^51^ or reward location^28^ have been previously reported. Specifically, it has been proposed that the grid cell system can anchor to displaced boundaries via a phase shift^52^. Adaptive geometric computational models have also suggested that grid computations can occur in distorted environments^53^. In these scenarios, the animal receives visual inputs from the surrounding environment. The phenomenon we report here differs in that the animal is required to perform a path integration-dependent homing task, where the lever location is unknown to the animal during dark trials. It has been shown that the absence of visual landmark inputs leads to grid pattern degradation over time due to error accumulation^8,9^. Previous reports indicate that when external landmarks are available, with sufficient navigational experience, grid firing patterns in separate environments, initially local, become globally uniform^54^. Consistent with these findings, we observed a stable grid pattern during random foraging trials when the lever was present in the arena. However, this grid pattern was not maintained in light trials of the AutoPI task, despite the lever being visible, that is, in the condition in which grid cells also anchor back to the room reference during homing. This dynamic reanchoring, even when external landmarks are available, points to the flexibility of grid cells in adapting to varying environmental contexts and navigation tasks.

During light trials, we observed high map stability in both the room and lever reference frames. Although the anchoring strength to the lever increased after encountering the lever, it remained significantly lower than that observed in dark trials. One possible explanation for these findings is that, in light conditions, grid cells flicker between reference points^28^. Thus, frequent changes in the reference system mask the grid pattern, and anchoring to either reference system is low, while map stability in both reference frames is maintained.

Our findings highlight that the absence of a grid pattern does not necessarily equate to a loss of grid cell functions. Single-cell analysis reveals that the grid cell firing pattern is modulated by the changing lever location, and maintains positional information relative to the bridge during search and to the lever during homing. This would suggest that after reanchoring a grid module to the lever, the information about the distance to the bridge in this module is lost. We do not know, however, if a similar reanchoring phenomenon takes place in larger grid modules. Although stable grid patterns were not observed during the AutoPI task, the temporal firing associations and phase relationships of population grid cells within a module remained preserved, even after a within-trial reanchoring event. This observation supports previous findings indicating that grid cell population activity spans a toroidal manifold that remains invariant across different environments and states^32,34^. Moreover, we could decode movement direction with a significant level of accuracy in spite of the absent grid pattern, demonstrating that grid cell modules reflected the path integration process within both the room reference frame and the current local reference frame.

Such consistency in the toroidal representation is important for path integration, as it ensures a stable mapping of local space onto this internal manifold. In our study, grid cells maintained stable phase relationships, and we could reliably decode integrated self-motion cues from the grid cell activity, which is essential for calculating the animal’s position relative to its starting point. These findings indicate that the absence of a stable grid pattern was due to the reanchoring of the grid phase when the animal reached the lever and noise accumulation in the estimation of orientation and position when navigating using self-motion cues in darkness^8,9,55^. However, whether path integration takes place within the grid cell network itself or upstream remains an open question.

We found that within a trial, the drift in grid cell orientation correlates with the mouse’s homing direction. Combined with positional analysis, these results show that while the distance coding is reset to code for distance to the lever, the orientation of the grid map is not, thus allowing for computations in a similar orientation across multiple reference frames. A close relationship between the head-direction system in the anterodorsal thalamus and homing vectors was reported in a different homing task in rats^31^. The anatomical colocalization of grid cells and head-direction cells in layer II of the MEC^56^, along with findings that inactivation of head-direction cells disrupts grid cell firing^57^, supports a close interplay between these two cell types. Nevertheless, molecular manipulations affecting grid coding but leaving intact head-direction coding suffices to impair path integration performance^19^. Interestingly, the modulation of grid cell firing rate by head direction did not contribute to the directional precision of our decoding in our recorded data and simulations. These results suggest that movement signals from population grid cells are likely integrated from both upstream head-direction inputs and self-motion signals. While the head-direction signal provides a directional cue in polar coordinates, it cannot support path integration by itself. The movement-driven signal decoded from the grid cell population encodes positional information within a given reference frame and reflects the path integration process across reference frames.

The computations performed by grid cells during memory or navigation tasks have been notoriously difficult to characterize because standard measurements of grid cell activity require recording periods of several minutes, whereas behaviorally relevant events often last only a few seconds. With our computational framework to monitor grid cell activity in toroidal space, we could infer the grid orientation and grid phase anchoring with a sub-second resolution. This opens up the possibility of characterizing the properties of grid cells at the module level during most standard navigation and memory paradigms by removing the need for long behavioral epochs with extended spatial coverage. This approach and similar module-level analysis^32,34,58^ will contribute to understanding grid cell functions in a broader spectrum of behaviors.

## Methods

### Subjects

All procedures followed the European Council Directive (86/609/EEC) and were approved by the Governmental Supervisory Panel on Animal Experiments of Baden Württemberg in Karlsruhe (G-236/20). The subjects were 17 5–9 months old C57BL6/N male mice from the Interfaculty Biomedical Facility of Heidelberg University, Germany. The mice were kept on a 12-h light—12-h dark cycle, with all experiments performed during the light period. The mice were single-housed in 26 × 20 × 14-cm plexiglass cages with food and water ad libitum. The cage contained 2–3 cm of sawdust and was enriched with paper tissues. Room temperature was around 23.2 °C and room humidity was around 52.9%. The experimenters handled the mice for 5–10 min, once or twice a day, for 3 days before the training procedure started. On the last day of handling, the body weight of the mice was recorded, and food intake was controlled to maintain the body weight at 85% of the initial recorded weight.

### Apparatus for the AutoPI task

The behavioral apparatus consisted of a home base and a circular arena, both elevated 45 cm above the floor. The home base was a rectangular box (20 × 30 × 30.5 cm) with a front-facing sliding door and a food magazine attached to its back wall. The food magazine was equipped with an infrared beam to detect the presence of the mouse at the magazine. Food rewards (AIN-76A Rodent tablets 5 mg, TestDiet) were delivered into the magazine from a pellet dispenser attached to the external side of the home base outside wall. The sliding door of the home base could be lowered to give access to a bridge (10 × 10 cm) leading to the arena.

The circular arena (diameter of 80 cm) was mounted on a tapered roller bearing to allow clockwise and anticlockwise rotations. Eight 1.6-cm-high wall inserts were attached to the edge of the arena. The space between adjacent inserts was 10 cm, creating eight evenly spaced openings that potentially led to the bridge. One of the eight openings always faced the home base bridge when the mouse was on the arena. The arena rotation, movement of the door and delivery of food rewards were operated via Arduino Uno microcontrollers, digital stepper motor drivers (Stepperonline, DM542T) and N17 stepper motors. Two cameras (above the home base and the arena) connected to a microcomputer (Jetson Xavier NX) were used to monitor the animal and lever during the task. The videos were captured at 30 Hz (640 × 480 pixels), analyzed online and stored for further offline processing.

A lever (13 × 10 mm) extended from one wall of an autonomous lever box (116 × 82 × 80 mm). The lever box contained two servo motors, an Arduino Nano connected to a radio-frequency module (NRF24L01) and a 2,000-mAh lithium battery. Pressing the lever broke an infrared beam inside the lever box. Lever-press time stamps were transmitted via the radio module to a microcomputer controlling the task (Jetson Xavier NX). The same radio module was used to instruct the lever box to move to a different location on the arena. The location and orientation of the lever box were obtained using DeepLabCut (https://github.com/DeepLabCut/DeepLabCut/). When moving the lever between trials, the intended future location and orientation were randomly selected within a circle centered on the arena center with a radius of 75% of that of the arena. The Arduino Nano inside the lever box was instructed to move along the vector ending at the new locations. The new position and orientation were confirmed via DeepLabCut and corrected if needed.

Trials in the AutoPI task were performed with and without visible light and were referred to as light and dark trials, respectively. During light trials, two LED stripes (30 cm, white) above the arena were the only visible light source. An Arduino equipped with a relay module turned them on and off. During dark trials, the only light sources were infrared light LED stripes and lamps (850 nm) positioned above the arena and the home base. A white noise (65 dB) generated from a speaker above the arena was played during the task to mask uncontrolled auditory cues.

A Python script running on a microcomputer (Nvidia Jetson NX) controlled the logic of the task. Modular computer programs were responsible for controlling the different elements of the task (door movement, arena rotation, visible light, lever position and food delivery). The Robot Operating System (https://www.ros.org/) established communication between these nodes. Task-related events (for example, lever press, food delivery, door movement, arena rotation, light switch) and their respective time stamps were saved into a log file.

### AutoPI task training

Training on the AutoPI task took approximately 2 months. Mice were first familiarized with the home base for 20 min daily for 3 days. A food reward was delivered every 30 s. The lever box was placed inside the home base with the mouse on the fourth day. From this point, food was delivered when the mouse pressed the lever. These daily sessions were terminated after 30 min or 100 rewards, whichever came first. The position of the lever inside the home base varied from day to day. After 6 days of lever training in the home base, the door of the home base was opened and the lever was placed on the bridge. Over subsequent days, the lever was 2–3 cm further away from the home base until it was located at the center of the arena. Once the lever was at the center of the arena, we trained the mice until they obtained 70 rewards within 30 min. Once this criterion was reached, door movements and arena rotations between trials were introduced. During this phase, the mice learned to press the lever independently of its orientation. White noise (65 dB) was also introduced at this point. Then, lever movements were performed every four trials, forcing the mouse to press the lever at different positions and orientations on the arena. The lever only moved between trials.

### AutoPI task

We performed 10–15 behavioral training sessions before the surgical procedure. These sessions included light and dark trials and ended after 60 min or when the mouse performed 100 trials, whichever came first. After the surgical procedure, the AutoPI sessions with electrophysiological recordings lasted approximately 90 to 120 min. The recording sessions took place in a different room than that in which the AutoPI task training took place.

Every trial on the AutoPi task started with the mouse in the home base and the opening of the door. The mouse then searched for a lever on the arena. Pressing the lever led to the delivery of a food pellet in the food magazine of the home base. The trial ended when the mouse reached the food magazine after pressing the lever or after 5 min, whichever came first. The mouse could perform several journeys on the arena during the same trial if the lever had not been pressed during this trial. The mouse was confined to the home base between trials.

Every testing session started with a consecutive seven trials under visible light (light trials) followed by an alternating sequence of dark and light trials. All sources of visible light were turned off during dark trials.

The arena was rotated after each trial to one of eight possible angles (multiple of 45°). The lever moved to a new random location on the arena every fourth trial. The combination of arena rotations and lever movement ensured that the lever moved to a new location relative to the home base in most trials. The arena floor and home base were cleaned with ethanol at the end of each recording session. The experimenter monitored the AutoPI session from a different room.

On the arena, the animals could see the blue arena borders interspersed with openings at regular intervals. The AutoPI setup was placed in a corner of the room, the arena being closest to the corner. Assuming that the home base pointed south, the animals would thus perceive the room walls in the north and west and the bridge leading to the home base in the south. The east direction was open toward the room. The mice could perceive the spare arena leaning upright against the wall in this direction. The AutoPI setup was placed within a metal frame, so that there would be metal bars in the northwest, northeast, southwest and southeast directions. In addition, when looking upward, the mice would perceive metal bars stretching along the north–south direction and cameras attached to them. While in the home base with the door closed, the mouse would only perceive the walls and the food magazine on the wall opposite the door. The cues were identical for all animals throughout the project.

To account for differing lever position probabilities in light and dark trials, we divided the trials into trials with and without lever changes and repeated the behavioral analysis. We found that unchanged lever positions did not affect the mouse’s search or homing behavior (Supplementary Fig. 12).

### Surgical procedure

Once the AutoPI task training was completed, the food restriction was paused. The mice were implanted with one H64LP NeuroNexus probe or one or two H10 Cambridge NeuroTech probes. Before implantation, the probes were mounted onto custom-made microdrives, allowing linear movement in the dorsoventral axis. Anesthesia was induced with isoflurane (1.5–3%), and the mouse was fixed to the stereotactic frame. Mice were kept under 0.5–1.5% isoflurane during the surgery. The skull was exposed, and two miniature screws were attached to the skull. One screw located above the cerebellum served as a ground/reference electrode. A small craniotomy was performed above the posterior sinus 3.1 mm from the midline. The probes were implanted 0.2 mm anterior of the transverse sinus at an angle of 6–7° in the sagittal plane, with the probe pointing down with a slight angle in the posterior direction. The shanks of the probes were lowered 0.6–0.8 mm into the brain, and the microdrives were fixed to the skull with dental cement. During the first 72 h after the surgery, the mice received Carprofen (0.1 mg per kg body weight Rymadil, subcutaneously) every 8 h. Mice were given at least 6 days of recovery before food restriction and training resumed.

### Recording sessions

Each recording session started with a 30-min random foraging trial, during which electrophysiological and positional data were collected as the animal explored a circular arena (diameter of 50 cm). The mouse was moved to a rest box (30 × 30 × 35 cm) for 10 min. The AutoPI task was then performed for 90 to 120 min. The mouse was then moved back to the rest box for 5 min. A second 30-min random foraging trial was conducted. Random foraging trials are used as a baseline to identify grid cells and compare grid cell properties during random foraging and the AutoPI task. In addition, we use the first random foraging trial to train a machine learning decoder and test it in the AutoPI task and the second random foraging.

After each recording session, the probes were lowered into the brain. For unilateral implantations, the probe was lowered by ~62.5 µm (NeuroNexus Probes) or ~125 µm (Cambridge Probes). For bilateral implantations, only one probe was lowered on a given day (~125 µm), alternating between the left and right probes.

On the final day of recording of each mouse, we performed additional recording trials. The lever was placed on the arena for a 15-min random foraging trial. During this trial, the mouse had no access to the home base of the AutoPI task. The lever’s location on the arena was changed after 15 min, and recordings continued for another 15 min.

### Histology

At the end of the experiment, the mice were deeply anesthetized using a solution of ketamine (20%; 50 mg ml^−1^) and xylazine (8%; 20 mg ml^−1^) injected intraperitoneally. Transcardial perfusion was performed with 4% paraformaldehyde. The extracted brains were stored in paraformaldehyde at 4 °C for 24 h. The brains were washed with phosphate-buffered saline and cut into 50-µm-thick sagittal slices with a vibratome (Leica). Cresyl violet staining was performed, and stained sections were digitized with a motorized widefield slide scanner (Axio Scan.Z1, Zeiss).

### Electrophysiological recordings, spike extraction and spike clustering

During electrophysiological recordings, the mouse was connected to a data acquisition system (RHD2000-Series Amplifier Evaluation System, Intan Technologies, analog bandwidth 0.09–7603.77 Hz, sampling rate 20 kHz) via a lightweight cable. The recording hardware was controlled using ktan software (https://github.com/kevin-allen/ktan/). Spike extraction and clustering were performed in a semiautomatized manner using Kilosort (https://github.com/jamesjun/Kilosort2/). Phy (https://github.com/cortex-lab/phy/) was used to refine the spike clusters manually.

Only spike clusters with a distinct waveform, a mean firing rate above 0.1 Hz and a refractory period ratio smaller than 0.15 were analyzed to ensure high cluster quality. The refractory period ratio was calculated from the spike-time autocorrelation from 0 ms to 25 ms with a bin width of 0.5 ms. The refractory period ratio was defined as the mean number of spikes in bins from 0 ms to 1.5 ms, divided by the maximum number of spikes in any bin between 5 ms and 15 ms. We excluded clusters with fewer than 100 spikes in their spike-time autocorrelation (from 0 ms to 30 ms).

### Recording cable actuator

During recording sessions, the online tracking data were fed to a motorized cable actuator, which ensured that the upper attachment point of the recording cable was kept above the head of the animal. The actuator minimized the interference of the recording cable with the mouse’s behavior. The cable actuator was positioned 153 cm above the arena, with a movement range of 85 × 130 cm in the horizontal plane.

### Position tracking and behavioral analysis

For AutoPI sessions without electrophysiological recordings, we tracked the mouse’s position on the arena with a DeepLabCut model that determined the position of the nose and ears of the mouse. The midpoint between the two ears was used as the animal position. For AutoPI sessions with electrophysiological recordings, an infrared LED (940 nm) array was attached to the implant and consisted of three LEDs on one side and one LED on the other side, with a distance of 3 cm between them. The mouse’s position was estimated online using OpenCV functions (for the cable actuator described below). The mouse’s position was recalculated offline using DeepLabCut (https://github.com/DeepLabCut/DeepLabCut/) or unetTracker (https://github.com/kevin-allen/unetTracker/).

Each AutoPI session was segmented into trials, which started when the door opened and ended when the door closed. The mouse’s path on the arena was divided into search and homing paths from when the mouse pressed the lever. The search path begins at the start of the journey and continues until the animal reaches the lever. When the mouse came within 10 cm of the lever box’s wall, it was considered to be at the lever. The homing path extends from the moment the mouse leaves the lever until it reaches the periphery of the arena. The mouse was considered to have reached the periphery of the arena when its position was less than 3 cm from the arena’s edge. Trials in which the lever was less than 10 cm from the arena border were excluded from the analysis.

We calculated the median heading direction of the animal during the search and homing paths. Two homing errors were calculated from the homing path—an error at the periphery and a heading error (Extended Data Fig. 2a). We calculated the error at the periphery by measuring the angle between the coordinates of the animal at the periphery to the center of the arena and the center of the bridge to the center of the arena. The heading error was defined as the median heading of the animal during homing until it reached the periphery.

### Identification of grid cells

Firing rate maps were constructed by dividing the area on the circular arena into 3 × 3-cm bins. The number of spikes and the seconds spent in each bin of the maps were calculated, resulting in spike count and occupancy maps. The spike count and occupancy maps were smoothed using a Gaussian kernel (standard deviation of 5 cm). A firing rate map was obtained by dividing the values of the spike count map by those of the occupancy map.

Grid scores were calculated from the spatial autocorrelation of the firing rate map. The location of the six fields surrounding the center of the spatial autocorrelation was obtained to define a circular region in the spatial autocorrelation containing the six fields but excluding the autocorrelation center. The selected region was rotated by 30°, 60°, 90°, 120° and 150°. The rotated regions were correlated with the original, non-rotated region. The grid score was defined $\frac{({r}_{60^\circ }+{r}_{120^\circ })/2}{({r}_{30^\circ }+{r}_{90^\circ }+{r}_{150^\circ })/3}$, where $r$ is the Pearson correlation coefficient between the non-rotated and rotated regions. The significance levels for grid scores were obtained in a cell-by-cell manner by calculating a grid score 500 times after shifting the position data by a random amount (minimum shift of 20 s). Grid cells were identified using the first random foraging trial and were defined as neurons with a grid score larger than the 95th percentile of the random grid score distribution.

A grid cell periodicity matrix was generated by performing a correlation between the rotated and non-rotated circular region of the spatial autocorrelation by steps of 2° (Fig. 2b). Each row of the periodicity matrix contains the correlation coefficient from one grid cell.

### Measurement of information score

The information score of each neuron’s firing rate histogram was calculated following previous literature^59^, as shown in equation (1):

1 $$\mathop{\sum }\limits_{{\rm{i}}=1}^{{\rm{N}}}{{\rm{p}}}_{{\rm{i}}}\frac{{{\rm{\lambda }}}_{{\rm{i}}}}{{\rm{\lambda }}}{\log }_{2}\frac{{{\rm{\lambda }}}_{{\rm{i}}}}{{\rm{\lambda }}}$$

Where *N* is the total number of histogram bins. *p*_*i*_ is the probability of occupancy in bin *i*, *λ* is the overall mean firing rate of the neuron and *λ*_*i*_ is the firing rate in bin *i*. Histograms were not smoothed before calculating information scores.

### Cluster isolation distance

The cluster isolation distance is the radius of the smallest ellipsoid containing all spikes from that cluster and an equal number of spikes from other clusters following ref. ^60^. For each cluster, spikes from all other clusters recorded on the same shank were considered as reference spikes. Spikes for the entire session were used.

### Measurement of firing association and spike-triggered cross-firing rate maps

Firing association quantifies the correlation between the instantaneous firing rates of pairs of neurons across a set of conditions. It is calculated by computing the Pearson correlation coefficient between the instantaneous firing rates of pairs of neurons. The temporal firing rate was calculated using a bin size of 100 ms and smoothing with a Gaussian kernel of sigma = 1 (100 ms) was applied.

To calculate spike-triggered cross-firing rate maps between pairs of grid cells A and B, the spikes of grid cell A served as reference spikes. For each reference spike of grid cell A, we calculated the position of the mouse at the time of spike of grid cell B *P*_*B*_ relative to the position of the mouse at the time of the reference spike *P*_A_, together with the time the animal spent at different locations relative to *P*_A_. We limited the analysis to a temporal window of 0.5 s centered on the reference spikes, and only bins within a radius of 10 cm from the center of the spike-triggered cross-firing rate map were kept. This resulted in a map containing the firing rate of grid cell B relative to the spike of grid cell A. For a shuffled control, we randomly permuted 200 times the cross-correlations of condition 1 and then averaged the correlations across animals.

### Reconstruction of movement path from grid cell activity

Recording sessions with at least five grid cells were used for movement path reconstruction. The reconstruction method involved transforming the Cartesian coordinate system into a toroidal coordinate system. This transformation depended on the orientation of axes of the grid pattern and their respective periods. We used a multistep approach to obtain the best estimate for these parameters. We estimated the peak firing rate and grid offset from the firing rate map. The orientation and spacing of the grid cell were obtained from the spatial autocorrelation. These parameters were optimized by fitting the instantaneous firing rate of each grid cell with two grid cell models. The models took the mouse’s trajectory in the open field as input and predicted the firing rate of the neuron for each position data point. The model had three grid axes with their respective direction referred to as ${\theta }_{0},{\theta }_{1},{\theta }_{2}$. The mouse’s position along the three grid axes was calculated (${d}_{0},{d}_{1},{d}_{2}$). These positions were transformed into angles (${a}_{0},{a}_{1},{a}_{2}$) using ${a}_{i}=\frac{{d}_{i}}{{p}_{i}}2\pi$, where *p*_*i*_ was the period of each grid axis. The grid pattern of the model had an offset in the three axes (${o}_{0},{o}_{1},{o}_{2}$). The firing rate of the simulated neuron was obtained according to equation (2):

2 $${\rm{ReLu}}((\cos ({a}_{0}-{o}_{0})+\cos ({a}_{1}-{o}_{1})+\cos ({a}_{2}-{o}_{2})+1.5)/4.5\times {\rm{pr}}),$$

where ‘pr’ is the peak firing rate of the neuron and ‘ReLu’ is a rectified linear unit function. The first model was constrained to have perfect 60° periodicity with axes direction of $\theta ,\theta +\pi /3,\theta +2\pi /3$, and equal axis periods (${p}_{0}={p}_{1}={p}_{2}$). In the second model, the axis directions were free to deviate from 60° periodicity, and the axis periods could differ. The estimated parameters of the first model were used to initialize the second model. The models were fitted using gradient descent, a mean squared error loss function, and an Adam optimizer. We fitted the two models sequentially to minimize the effect of local minima during gradient descent.

We used the median of ${\theta }_{0},{\theta }_{1},{p}_{0},{p}_{1}$ of the simultaneously recorded grid cells as parameters to transform the Cartesian coordinate system into a toroidal coordinate system. The animal’s path on the arena was transformed into a path on the surface of a torus. For each position data point, the position (${d}_{0},{d}_{1}$) along the first two grid axes was obtained and transformed into angles ${v}_{0},{v}_{1}$ using ${v}_{i}=\frac{{d}_{i}}{{p}_{i}}2\pi$. After the transformation, the path of the animal in toroidal space was represented by the angles ${v}_{0},{v}_{1}$.

We used a multilayer long short-term memory RNN to decode the animal position in toroidal space from the instantaneous firing rate of grid cells. The network had two layers and 256 features in the hidden layers. We used the instantaneous firing rate of grid cells as input (bin size of 20 ms, smoothing kernel standard deviation of 20 ms). The model input was a matrix containing the firing rate of grid cells during the last 400 ms (20 bins), and the model output was a single position on the torus. To deal with the circularity of the coordinates on the torus, the model was trained to predict the cosine and sine of each angle: $\cos ({v}_{0}),\sin ({v}_{0}),\cos ({v}_{1}),\sin ({v}_{1})$. The two angles were obtained using $\arctan 2(\sin ({v}_{0}),\cos ({v}_{0}))$ and $\arctan 2(\sin ({v}_{1}),\cos ({v}_{1}))$. The model was trained on the first 80% of the first random foraging trial. The last 20% were used to test the model using data from the same trial. The model was trained for one epoch, using a batch size of 64, a learning rate of 0.001, a mean squared error loss function and an Adam optimizer. The decoded $\cos ({v}_{0}),\sin ({v}_{0}),\cos ({v}_{1}),\sin ({v}_{1})$ were smoothed using a Gaussian kernel (standard deviation of 100 ms).

The sequence of decoded positions in toroidal space was used to calculate the decoded movement path of the mouse in Cartesian space. We first calculate the difference Δ*v*_0_ and Δ*v*_1_ between adjacent *v*_0_ and *v*_1_ data points, representing the angular movement along the two dimensions of the torus. Δ*v*_0_ and Δ*v*_1_ were mapped into movement in centimeters along the two axes using ${\frac{\varDelta {v}_{i}}{2\pi }p}_{i}$. The movement along the two grid axes was then transformed into a movement along the *x* and *y* axes of the Cartesian coordinate system.

To assess path integration within the grid module, we calculated the decoded directional error, defined as the angle between the decoded movement vector and the animal’s real movement vector. From the distribution of decoded directional error, we extracted two parameters. Directional precision was the MVL of the distribution. The circular mean of the distribution represented the orientation of the grid module relative to the first random foraging trial (data used to train the RNN). To correct for the rotation of the grid module between the first random foraging trial and the AutoPI task, we subtracted the circular mean of the distribution from the grid orientation and reran the decoder. For each session, we calculated an average grid rotation angle for all trials of a particular type (light and dark trials, separately). The same rotation was applied to all trials of a given light condition within one session. We refer to these two rotations as session-wise rotational corrections (one for light and one for dark trials). The corrected decoded movement vectors were used for further analysis (Supplementary Fig. 13). A step-by-step explanation to our method is provided in Supplementary Video 1.

For the trial-by-trial grid orientation correction analysis, we correct for axes of the torus by applying corrections to each trial. Specifically, for each trial, the average grid rotation angle is calculated from the circular mean of the distribution of decoded directional error and subtracted from the grid orientation.

To quantify model performance, we compared the directional precision of the model’s decoding with a shuffled control. For the shuffled control, we randomly shifted the decoded movement vectors by at least 20 s 1,000 times and then computed the MVL for the distribution of the angle between these shuffled decoded movement vectors and the actual movement of the animal. The upper 95th percentile value is used for shuffle control comparison.

Grid cell movement decoding was attempted when at least five co-modular grid cells were recorded (49 sessions from 11 mice) because fewer grid cells produced decoding results near chance level. The model was trained with the data from the first random foraging trial and tested on the data from the second random foraging trial. Only sessions in which the directional precision on the test set was above 0.5 were included for subsequent decoding analysis (24 sessions). Because animals tend to move along the arena borders during random foraging, we excluded border data when testing model performance to ensure that decoding accuracy remained reliable for behaviors on the arena center. Additionally, we included spiking activity in the model analysis only when the animal was moving on the arena (>10 cm s^−1^). Exclusion criteria were established during the RNN model development but before data analysis in the AutoPI task.

### Measurement of grid module anchoring strength

To estimate the location of the lever in toroidal space, we calculated a Cartesian vector between the mouse and the lever, transformed this vector into toroidal space, and added the vector to the predicted location of the mouse in toroidal space (Extended Data Fig. 10b). This transformation includes rotating the vector by the session-wise rotational corrections (one for light trials and one for dark trials). To quantify the concentration of the inferred lever positions in toroidal space, we fitted a bivariate von Mises distribution^61^ to the inferred lever position. The parameters *k*_0_ and *k*_1_ of the bivariate von Mises distribution represent the concentration of the distribution. *k* = 0 for uniform distributions, and larger *k* values are observed for more concentrated distributions. The mean of the two concentration parameters served as the anchoring strength score. A similar analysis was conducted to assess the anchoring of the grid modules relative to the room reference frame. In this case, the bridge was used as the anchoring point. For the anchoring strength analysis presented in Fig. 6g and Extended Data Fig. 10f,g, the time spent in each condition (search, lever, homing) was divided into three equal segments, corresponding to the beginning, middle and end of the path. To standardize across trials, the maximum path duration for each condition was capped at 3 s.

To generate a shuffled anchoring strength when the animal is at the lever, we randomly sampled two values from a uniform distribution between -pi to pi for each trial in the AutoPI task. These two values are added to the predicted *v*_0_ and *v*_1_ coordinates decoded by the model for each trial when the animal is at the lever. Thus, for each trial, the reconstructed toroidal path was translated to a random position on the torus. The Cartesian vector from the real position of the animal pointing toward the real position of the lever at each time point is transformed into toroidal space and added to the shuffled *v*_0_ and *v*_1_ coordinates for the same time point to generate the shuffled lever position. To calculate the shuffled anchoring strength, we fitted a bivariate von Mises distribution to represent the concentration of the distribution, as previously described. This shuffling is performed 1,000 times to generate the shuffled anchoring strength distribution.

The temporal dynamics of anchoring strength as a function of time rise sharply after encountering the lever and peaking at around 1.6 s (Supplementary Fig. 14). It is important to note that there might be some variability between the moment we consider that the mouse reached the lever and the moment that the mouse perceived the lever. Unfortunately, we could not analyze single trials to determine if anchoring occurs rapidly at varying times per trial (our hypothesis) or develops over 1–2 s.

### Lever-centered firing rate analysis

We calculated firing rate maps in the lever reference frame. To obtain the position of the animal relative to the lever position, we subtracted the position of the lever from the animal’s position. Firing rate maps were then generated using a procedure similar to that of standard firing rate maps but the bin size varied (standard maps bin size, 3 × 3 cm; lever reference frame maps bin size, 1 × 1 cm) and only data points when the mouse was within a radius of 18 cm from the center of the lever were included. When these maps were compared to room referenced maps, room referenced maps were calculated using the same time intervals as lever-referenced maps. To calculate the map stability during light or dark trials, the trials were divided into two equally sized groups of trials, two firing rate maps were generated, and the Pearson correlation coefficient was calculated between the two firing rate maps.

Directional selectivity around the lever was estimated by calculating the firing rate of a neuron as a function of the direction of the mouse relative to the center of the lever. The direction of the mouse around the lever was defined as the direction of a vector with the origin at the center of the lever and pointing toward the mouse. The firing rate histogram had a bin size of 10°, and only data when the mouse was within a radius of 18 cm from the lever were included. The directional selectivity was defined as the MVL of this histogram. To calculate the MVL, a 2D vector was obtained for each histogram bin by multiplying the cosine and sine of the angle associated with a bin by the firing rate of the same bin. The 2D vectors were summed and divided by the sum of the firing rate in the histogram. The length of the resultant vector was the MVL. To estimate chance levels, directional selectivity was calculated after shifting the position data relative to the spike trains by a random amount (minimum shift of 20 s). This procedure was repeated 100 times, and the median was taken.

### Trial matrix of neuron firing rate

We created trial firing rate matrices to analyze the firing behavior of individual neurons on individual trials. Each matrix represented the firing rate of a single neuron. Rows in a matrix represented individual trials. The *x* axis represented different behavioral variables such as the distance traveled (bins of 2 cm), the *y*-axis coordinate of the animal position (bins of 2 cm), the distance of the animal to the lever (bins of 2 cm), time traveled (bins of 0.15 s) and the animal’s direction relative to the center of the lever (bins of 10°). A smoothing Gaussian kernel (standard deviation of two bins) was applied to each row of the trial matrices.

### Trial matrix of decoded directional error

We constructed trial matrices with the distribution of the decoded directional error for each trial (bins of 10°). The data in the matrices could be limited to the search path, the path at the lever or the homing path. A smoothing Gaussian kernel (standard deviation of 10°) was applied to each row of the trial matrices. Each row was normalized so that the highest value was 1. A white circle designates the circular mean of the decoded directional error within a trial. This circular mean of the distribution of the decoded directional error was referred to as the decoded trial drift.

### Trial matrix correlation

The trial matrix correlation was defined as the mean of the correlation matrix of all possible pairs of single trials of a session (rows of the trial matrix), excluding the unit diagonal. It indicates the similarity of the trials with regard to the variable analyzed in the trial matrix.

### Distribution of circular correlation coefficient

For each of the different conditions (search, at lever and homing), we calculated the circular correlation coefficient *r* between the trial directional drift and the homing heading angle in the corresponding trial and compared it to a distribution of *r* values expected by chance. Sessions exceeding chance level (above the 97.5th percentile) were considered significant.

### Statistics and reproducibility

Wilcoxon signed-rank tests, paired *t*-tests and Mann–Whitney rank tests were used to compare two groups. We used Kolmogorov–Smirnov tests to compare the shape of two distributions. For repeated measures, we used the Friedman test to assess differences. For pairwise correlations in which cells appeared several times, we used a crossed mixed-effects model. Circular–circular correlations were performed as previously described^62^. Circular distributions were compared using the Kuiper two-sample test. When appropriate, we used a shuffling procedure to establish significance levels. For paired Pearson correlation comparisons between two conditions, we report median *r* values and combine *P* values using Tippett’s method. The sample size was determined based on previous studies^19,30^. The investigators were not blinded to allocation during experiments and outcome assessment.

### Reporting summary

Further information on research design is available in the Nature Portfolio Reporting Summary linked to this article.

## Online content

Any methods, additional references, Nature Portfolio reporting summaries, source data, extended data, supplementary information, acknowledgements, peer review information; details of author contributions and competing interests; and statements of data and code availability are available at 10.1038/s41593-025-02054-6.

## Supplementary information

Supplementary Information Supplementary Figs. 1–14 and Supplementary Table 1.

Reporting Summary

Supplementary Video 1 Step-by-step explanation of our decoding methodology. This video provides a detailed, step-by-step walkthrough of the moment-to-moment decoding methodology used in our study.

Supplementary Video 2 Decoding of movement direction from grid cell activity. The instantaneous firing rate of simultaneously recorded grid cells served as input of an RNN. The RNN had previously been trained to predict the position of the mouse in grid toroidal space. The sequence of predicted toroidal positions forms a movement path in toroidal space. At each time point, the movement of the mouse in toroidal space is represented by a vector. This vector can be transformed into a vector in Cartesian space. The movement path of the mouse in Cartesian space can be reconstructed by summing the Cartesian movement vectors of the mouse. The real and predicted movement paths can be compared to assess path integration in grid cell modules.

## Source data

Source Data Figs. 1–8 and Extended Data Figs. 1–10 Statistical source data.

## Data Availability

The data generated in this study have been published on the Dryad platform under accession 10.5061/dryad.f7m0cfz80. Data frames used to generate the figures are provided in the Supplementary Information. Source data are provided with this paper.

## Code availability

The code used in this study can be found in GitHub repositories via:

https://github.com/PJJ19/autopi_grid_cell_decoding/,

https://github.com/PJJ19/Peng_et.al_2025_noInt/,

https://github.com/kevin-allen/autopipy/ and

https://github.com/kevin-allen/spikeA/.

Other packages used for this study include: scipy 1.11.3, scikit-learn 1.5.0, seaborn 0.12.2, statannotations 0.6.0, deeplabcut 2.2.3, pytorch 2.3.1, numpy 1.26.4 and pandas 1.5.3.
