# Supplementary material for: Grid cells accurately track movement during path integration-based navigation despite switching reference frames
Source: Nat Neurosci. 2025 Sep 10;28(10):2092–105. doi: 10.1038/s41593-025-02054-6 (PMC12497648; doi:10.1038/s41593-025-02054-6)
Supplement: Supplementary file 1 — Supplementary Figs. 1–14 and Supplementary Table 1. [file 41593_2025_2054_MOESM1_ESM.pdf]

nature neuroscience

Supplementary information https://doi.org/10.1038/s41593-025-02054-6

Grid cells accurately track movement during path integration-based navigation despite switching reference frames

In the format provided by the authors and unedited

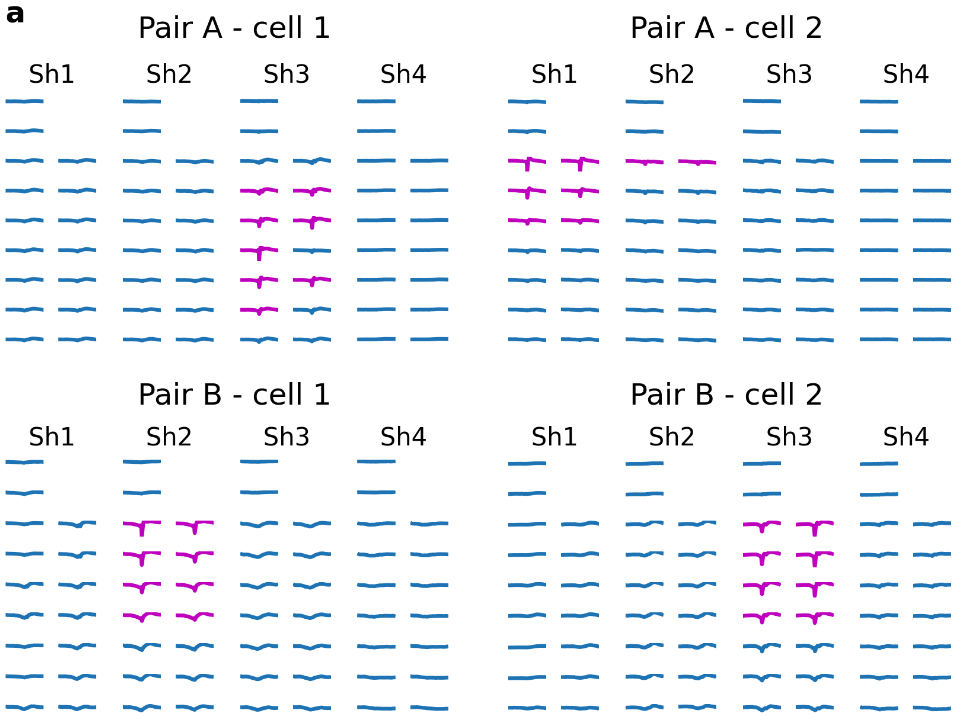

Figure S1: Example waveform between grid cell pairs recorded on different probe shanks
a, Spike waveforms in the approximated probe geometry (H64LP NeuroNexus probes) of the two grid cell pairs with the highest Pearson r correlations (Pair A: r in RF1 0.86, Pair B: r in RF1 0.83). The channels with the highest amplitude are colored in purple. Each probe shank (Sh1 - Sh4) has two rows of 7 electrode sites plus 2 single sites above.

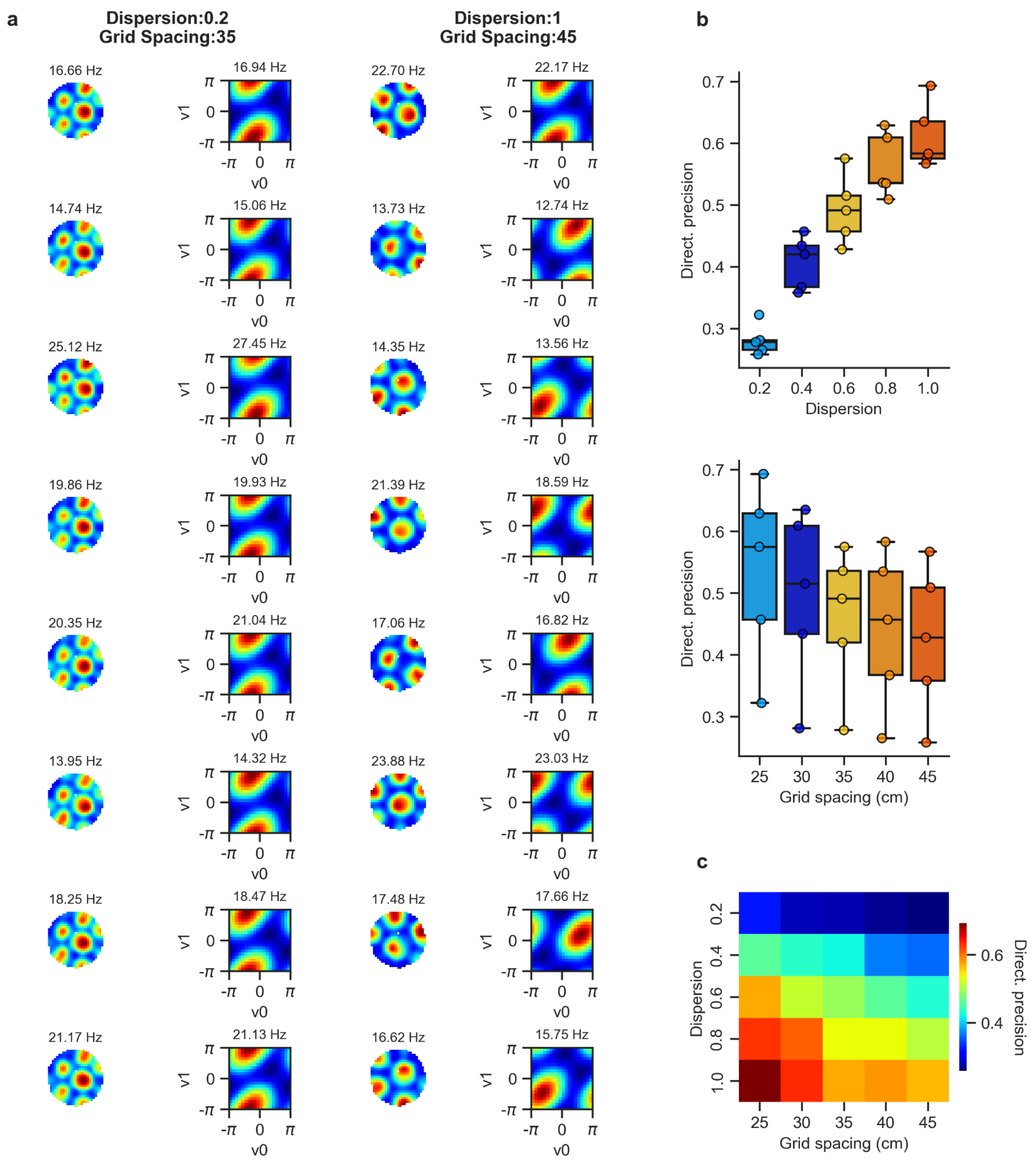

Figure S2: Impact of grid spacing and toroidal peak location dispersion on model decoding accuracy. **a**, To assess how grid dispersion and grid spacing affect model performance we simulated 8 grid cells. The simulation was repeated using different grid field dispersion and spacing. Grid field dispersion is defined as the spatial variability in the peak locations of grid cells on the torus, where higher dispersion indicates a wider spread of peak locations on the torus. For dispersion = 0, all grid peaks are concentrated at a single random location on the torus. For dispersion = 1, grid peaks are uniformly distributed across the toroidal space. Intermediate values of dispersion (0 < dispersion < 1) represent varying degrees of clustering around a random center on the torus, with higher dispersion values leading to broader spreads. Grid spacing refers to the average distances between 6 autocorrelation peaks and the center. First column: a group of 8 grid cells with grid dispersion of 0.2, and grid spacing of 35 cm, Second column: a group of 8 grid cells with grid dispersion of 1.0, and grid spacing of 45 cm. For each column, the left panel displays the firing rate map, and the right panel shows the corresponding firing rate map in toroidal space. **b**, Top: Directional precision of the model is assessed using grid cells with different grid field dispersion. Bottom: Model directional precision with different grid spacings. **c**, Heatmap of directional precision of the model assessed with different combinations of grid dispersion and grid spacing.

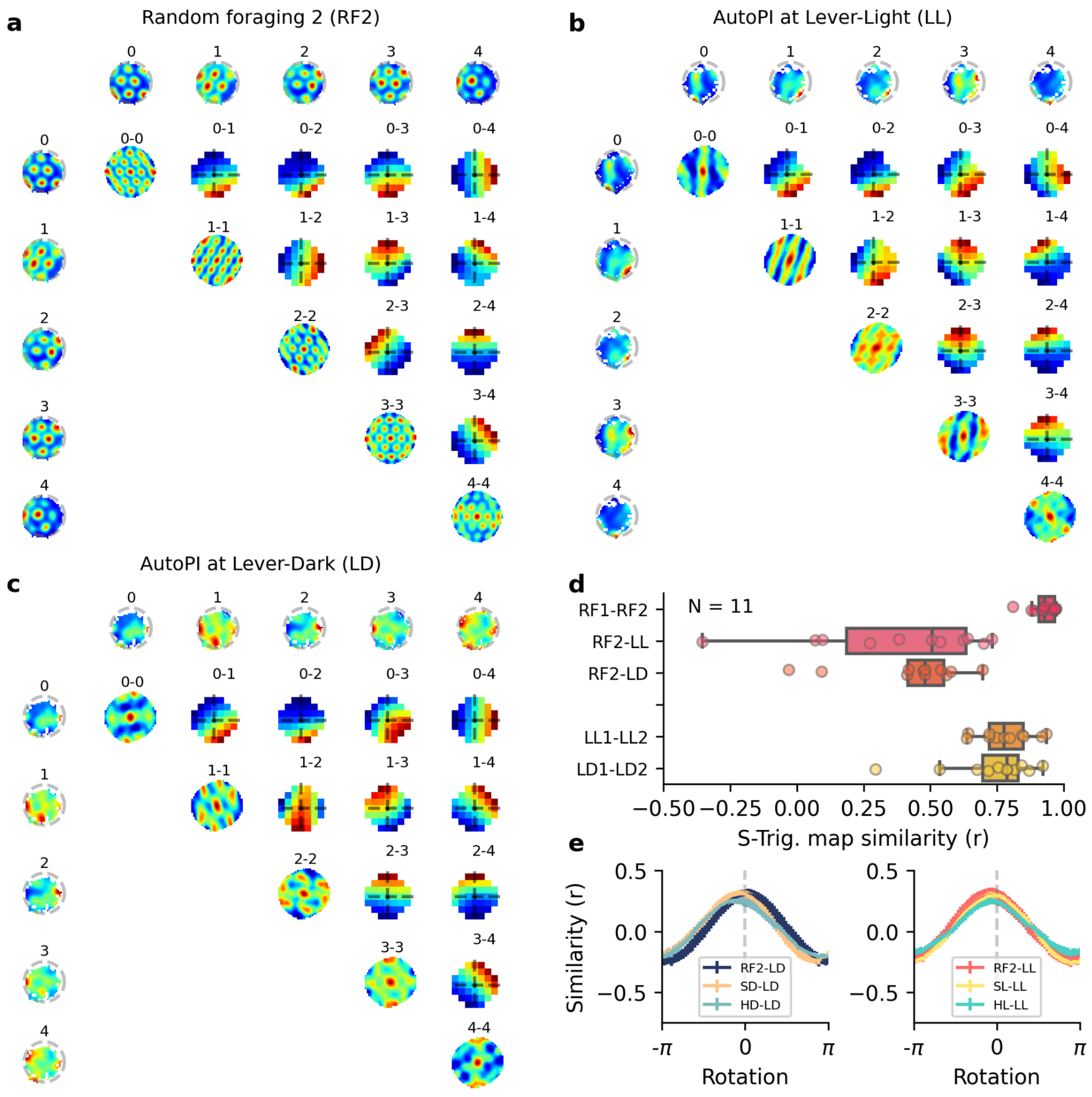

Figure S3: Preserved spatial offsets of grid cells at the lever location.
a-c, Spike-triggered cross-firing rate map between pairs of grid cells in random foraging (**a**), at Lever-Light (LL, **b**), and at Lever-Dark (LD, **c**). 5 co-recorded grid cells are shown for these three conditions. The column of maps on the left and the upper row of maps correspond to the firing rate map of these grid cells in random foraging (**a**), at Lever-Light (**b**) or at Lever-Dark (**c**). Autocorrelations are shown in the diagonal of the figure. The method for spike-triggered cross-firing rate maps are described in **Fig 3e**. The remaining maps are the spike-triggered cross-firing rate maps of each grid cell pair. **d,** Spike triggered map similarity (r-value) of grid cells across different conditions. The dots are the medians of individual mice (11 mice with at least 10 grid cell pairs; RF1-RF2: median r-value=0.923, P=2.117 x 10^{-33}, (Tippett's Method), P=9.766 x 10^{-4} (one-sample Wilcoxon signed-rank test); RF2-LL: median r-value=0.507, P=7.928 x 10^{-20}, (Tippett's Method), P=6.836 x 10^{-3} (one-sample Wilcoxon signed-rank test); RF2-LD: median r-value=0.481, P=2.032 x 10^{-25}, (Tippett's Method), P=1.953 x 10^{-3} (one-sample Wilcoxon signed-rank test); LL1-LL2: median r-value=0.777, P=4.545 x 10^{-28}, (Tippett's Method), P=9.766 x 10^{-4} (one-sample Wilcoxon signed-rank test); LD1-LD2: median r-value=0.788, P=3.348 x 10^{-27} , (Tippett's Method), P=9.766 x 10^{-4} (one-sample Wilcoxon signed-rank test). **e,** Rotation-correlation curves of map similarity between different conditions in the task. The similarity is assessed by rotating one condition and plotting the correlation with the other condition.

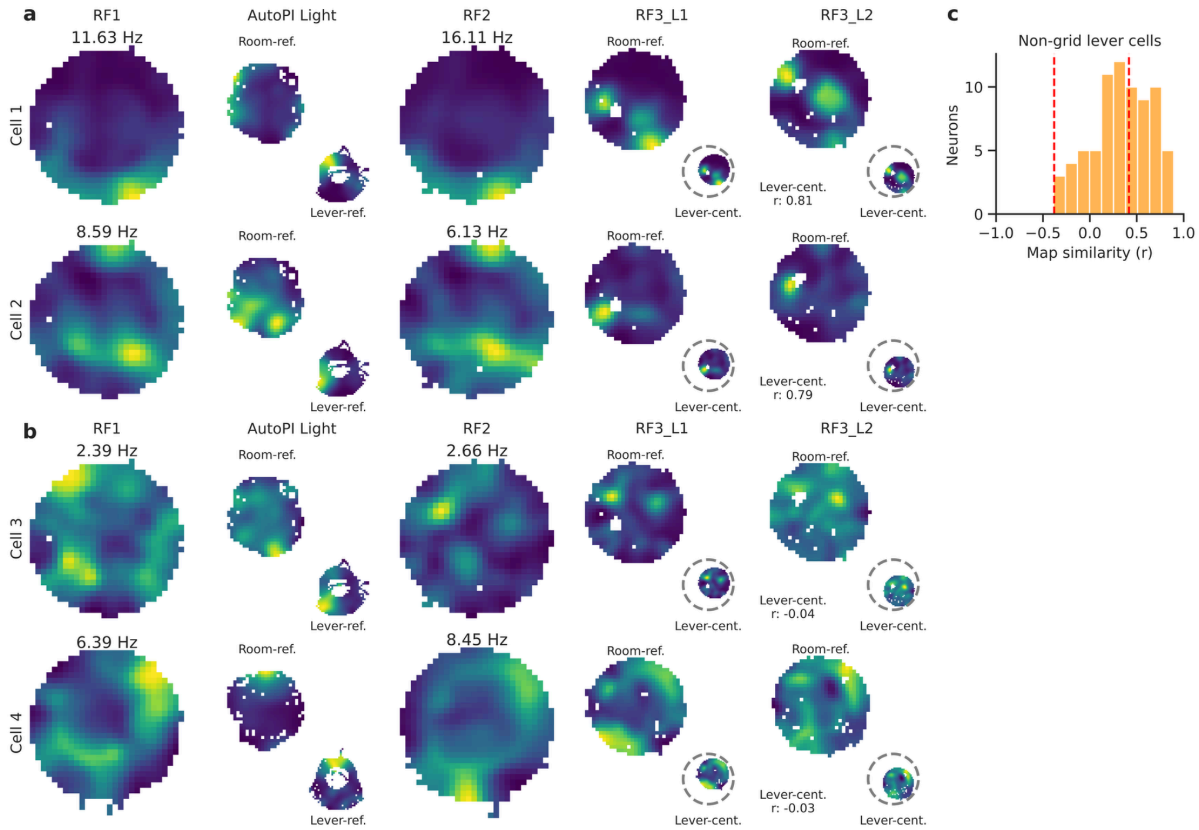

Figure S4: Differential firing rate maps of non-grid cells in task-relevant and task-irrelevant lever conditions.

a-b, Firing rate maps of four non-grid cells during the different conditions (random foraging (RF) and AutoPi task). RF3_L1: random foraging task with the lever on the arena. RF3_L2: random foraging task with the lever moved to a different location on the arena. In the bottom right panel, the firing rate map for AutoPI Light in the lever reference frame (see Methods) is presented. For RF3_L1 and RF3_L2, the lever-centered firing rate map is presented in the bottom right panel. Examples for object-dependent cells (**a**) and task-dependent cells (**b**) are shown. Object-dependent cells are cells with fields around the lever in the AutoPI task that show a lever-centered map similarity above significant shuffling threshold. Task-dependent cells are characterized as cells with fields around the lever in the AutoPI task but demonstrate lever-centered map similarity below the significant shuffling threshold. These cells showed low correlation between RF3_L1 and RF3_L2. **c**, Distribution of lever-centered map similarity between RF3_L1 and RF3_L2 for non-grid cells with fields around the lever. The red line denotes the 95th percentile of a distribution by chance (shuffling 1000 times). 41.9% of 74 cells exceed the 95th percentile of the shuffling distribution (red line).

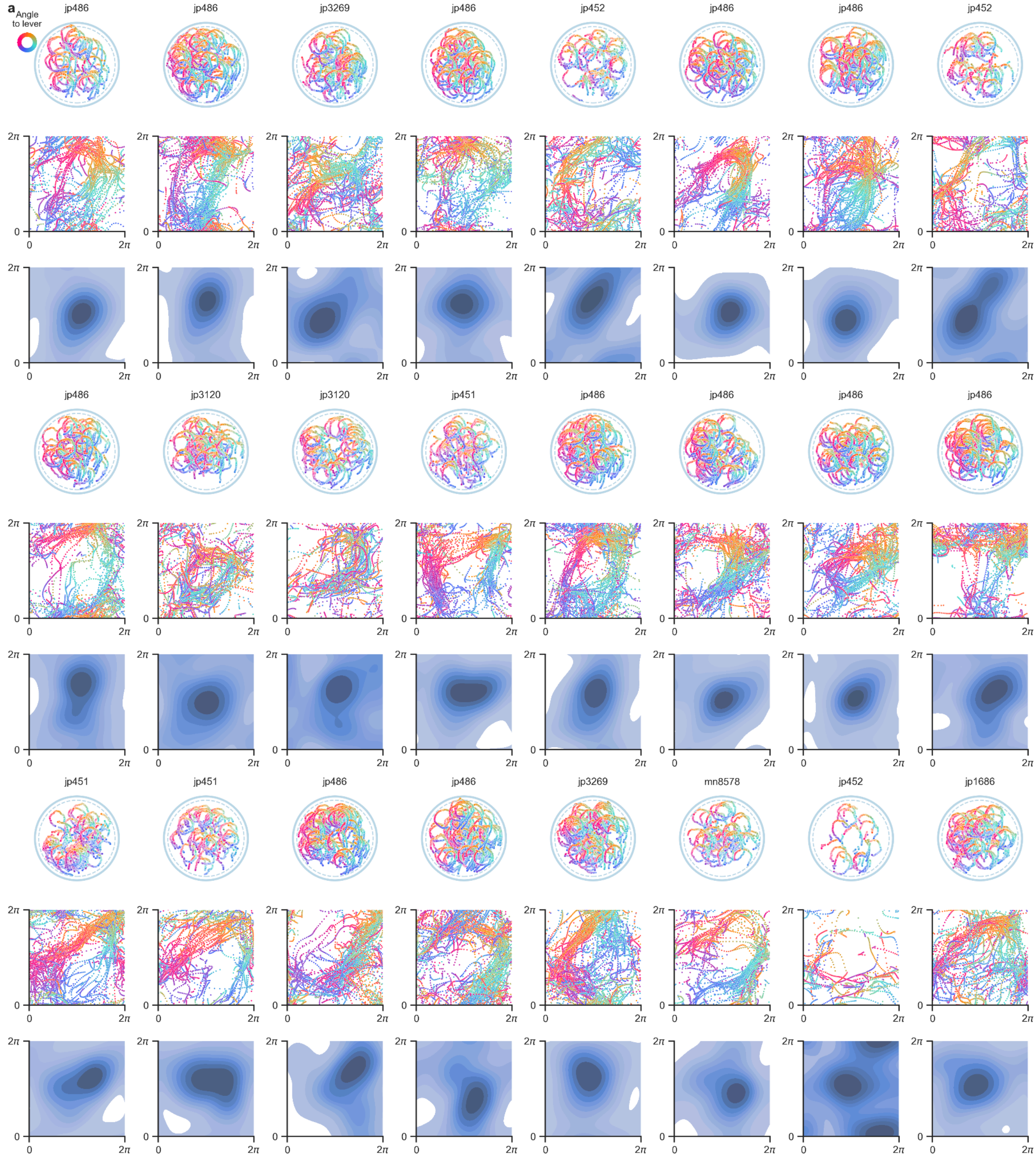

Figure S5. Anchoring of grid cell modules to the lever position during dark trials.

a, Extension to Figure 6d. All 24 sessions used for the reconstruction are presented. Sessions are sorted by the number of co-recorded grid cells. For each subpanel: Top: Examples of the running paths of the mouse around the lever during different recording sessions (Mouse running speed > 10 cm/s). The mouse's path is color-coded to reflect the direction of the mouse around the lever. Middle: Decoded position of the mouse in toroidal space when the mouse is around the lever. The color code represents the direction of the mouse relative to the lever. Bottom: Kernel density estimate of the decoded lever position in toroidal space when the mouse is near the lever.

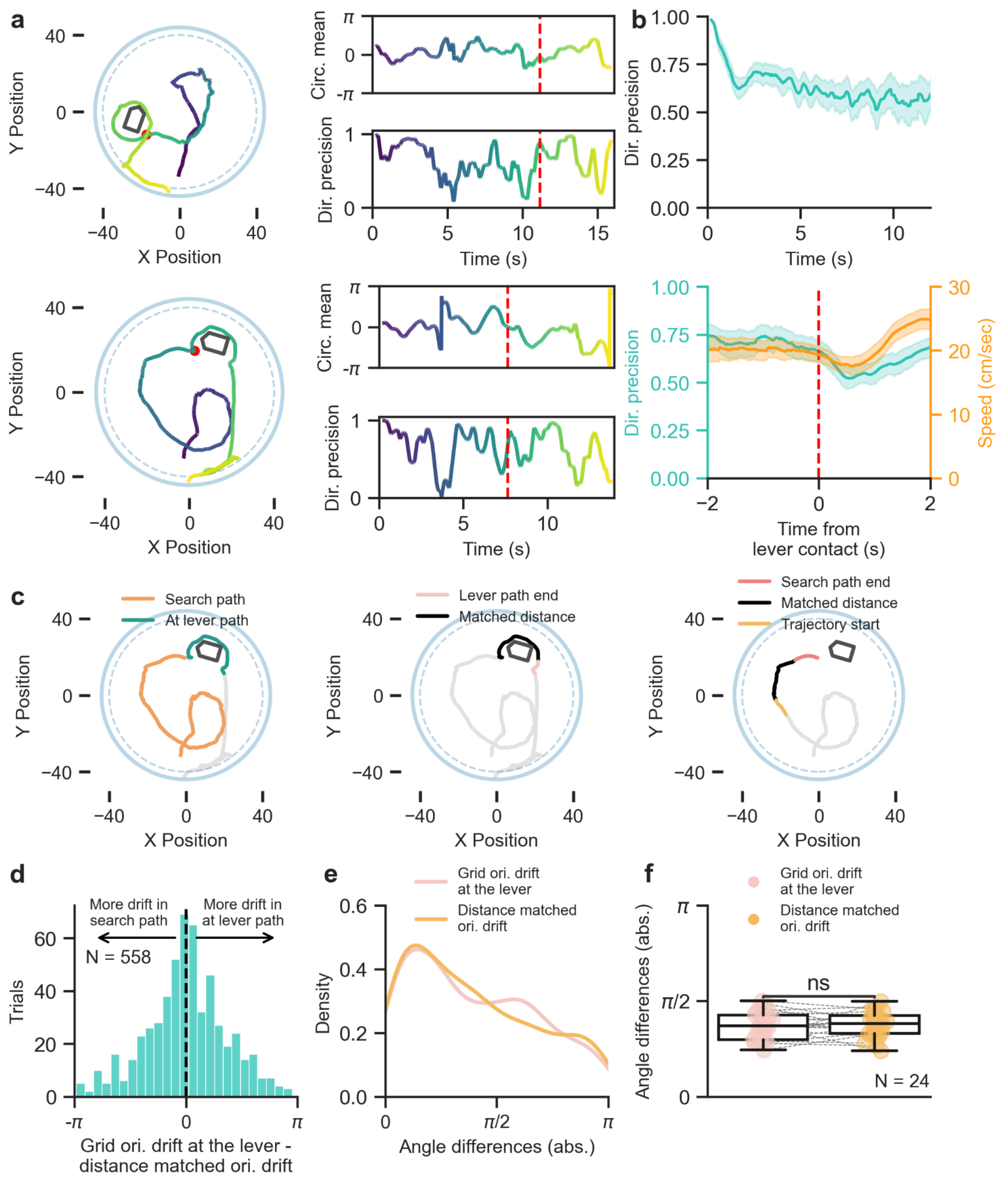

Figure S6: Encountering the lever during dark trials does not lead to more directional drift in the grid cell activity.
a, Two example dark trials, for each subpanel: Trajectory of the animal color coded by path time (left), red dot indicates the time of lever contact. Right, top: Circular mean of the moving average of the decoded direction error distribution of the trajectory to the left (bin size = 1s). Right, bottom: Directional precision (MVL) of the moving average of the decoded direction error distribution of the trajectory to the left (bin size = 1s). Dashed red line indicates the time of lever contact. **b**, Top: Directional precision (MVL) of the moving average of the decoded direction error distribution of all dark trials in the same session as the examples in a (right). Time 0 is the start of each trial. Bottom: Similar to the top figure but each trial is centered on the time of lever contact. 2 seconds before and after lever contact is shown for directional precision (left axis). For comparison, the orange line (right axis) shows the corresponding moving average (bin = 1s) of speed centered on the time of lever contact. **c**, Left: The search path of the trial in (**a**, second example) is shown in orange. The path of the animal at the lever is shown in green. The remaining path of the trial is shown in gray. The endpoint of the search trajectory is the end

of the search path in darkness. Middle: Example of the same trajectory. End of the lever path is calculated as the last quarter of the trajectory of the animal at the lever. Right: Example of the same trajectory. End of the search path is the trajectory of the animal during search, right before reaching the lever, distance-matched to the length of end of the lever path. The absolute difference between the circular mean of the decoded directional error for the end of the search path and the end of the lever path is called grid orientation drift at the lever. This drift represents the sum effect of two components: the direct effect of encountering the lever and the contribution of random, unbiased drift related to the distance traveled. To isolate the effects of the lever, we compared the grid orientation drift at the lever to a distance-matched orientation drift computed from search behavior on the same trial with no lever involved. Distance-matched orientation drift is calculated as the absolute difference between the circular mean of the decoded directional error for the end of the search path (red line) and the start of a distance-matched trajectory (orange line) on the same search path (matched distance shown in black). **d,** Distribution of the difference between grid orientation drift at the lever and distance-matched orientation drift for each trial. Only trials with a matchable search path distance were included (N=558 trials across 24 sessions, one-sample Wilcoxon signed-rank test, P=0.557). **e**, Distribution of grid orientation drift at the lever and distance-matched orientation drift in dark trials. **f**, Comparison of grid orientation drift at the lever and distance-matched orientation drift (N = 24 sessions, Wilcoxon sign-rank test, $P = 9.664 \times 10^{-1}$).

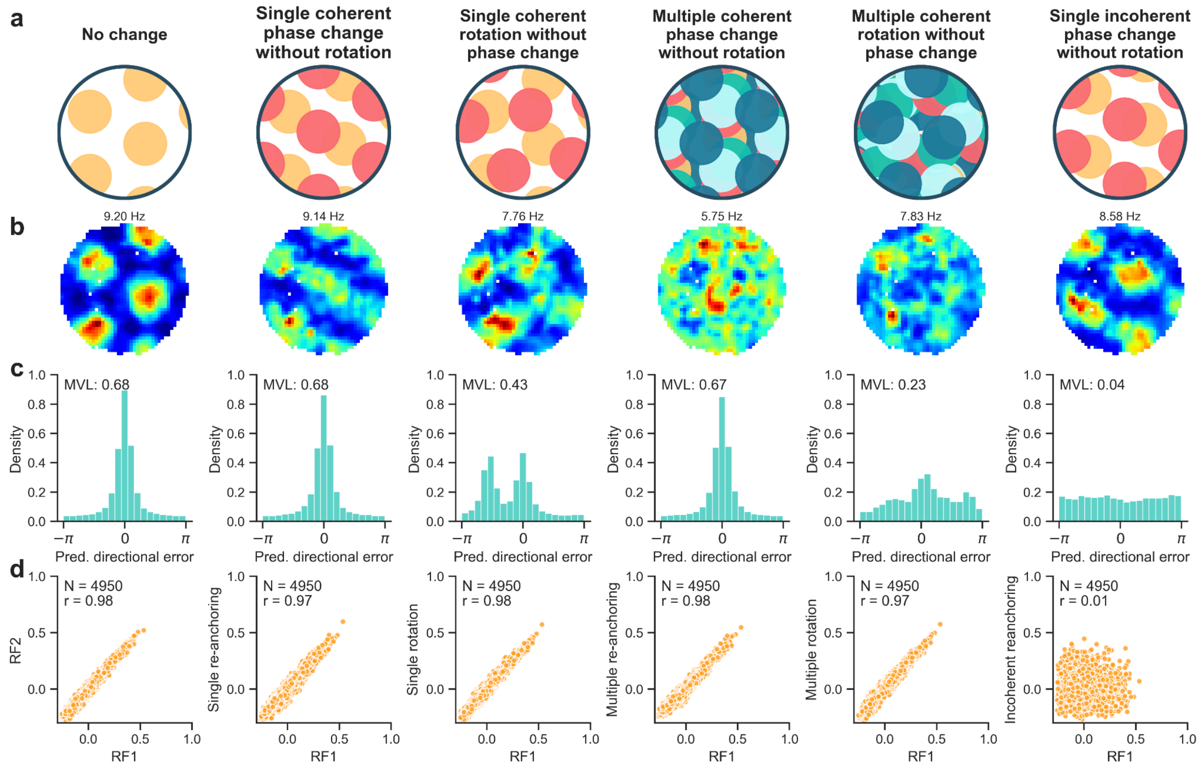

Figure S7: Decoding population grid cell activity in the absence of grid pattern.
a, Graphical abstract sequentially depicting six scenarios: (1) Unchanged phase and orientation across all grid cells; (2) Uniform phase shift for all grid cells without orientation change; (3) Uniform rotation in all grid cells without phase change; (4) Multiple uniform phase shifts; (5) Multiple uniform rotations; (6) Random translations without orientation change in each grid cell. **b,** Firing rate map of one grid cell in scenarios described in (A). **c,** Distribution of decoded directional error across the six scenarios. The mean vector length (MVL) of this distribution reflects the directional precision of the model. The circular mean of this distribution represents the rotation of the grid representation relative to the orientation of the grid cell during model training (first random foraging). **d,** Firing associations of all simulated grid cell pairs (N=4950) in each of the six scenarios.

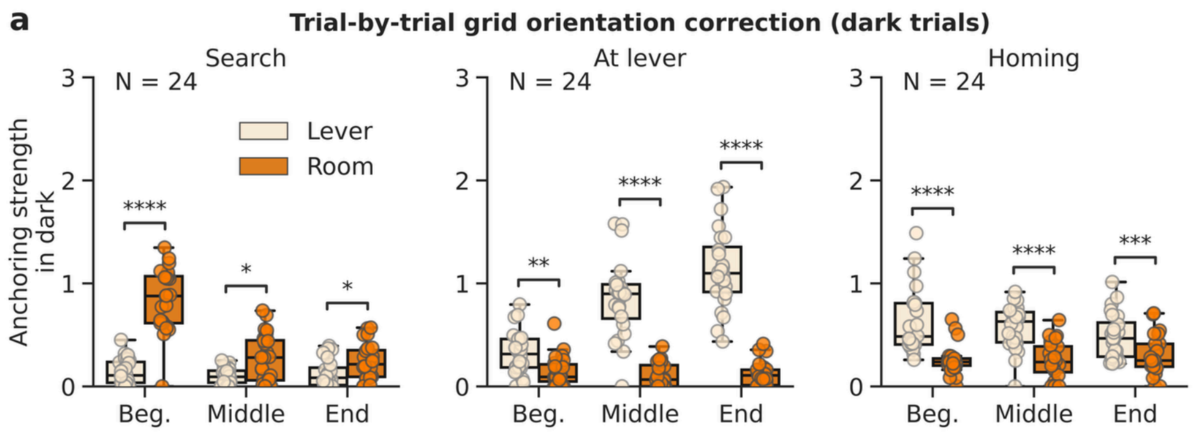

Figure S8: Grid module transitioned to the lever reference frame after the mouse reached the lever.
a, Anchoring strength to the lever or the room reference during dark trials, corrected for trial-by-trial grid orientation by aligning the axis of two axes of the torus with the grid rotation for each trial. Data during the search behavior, when the mouse was at the lever, and homing are shown separately and split evenly into the beginning, middle, and end (N = 24 sessions, Wilcoxon sign-rank test, Search, Beg.: $P = 2.384 \times 10^{-7}$, Middle: $P = 1.051 \times 10^{-2}$, End: $P = 2.440 \times 10^{-2}$; At lever, Beg.: $P = 1.780 \times 10^{-3}$, Middle: $P = 2.384 \times 10^{-7}$, End: $P = 1.192 \times 10^{-7}$; Homing, Beg.: $P = 3.016 \times 10^{-5}$, Middle: $P = 6.390 \times 10^{-5}$, End: $P = 6.498 \times 10^{-4}$). ****$P < 0.0001$, ***$P < 0.001$, **$P < 0.01$, * $0.01 < P < 0.05$.

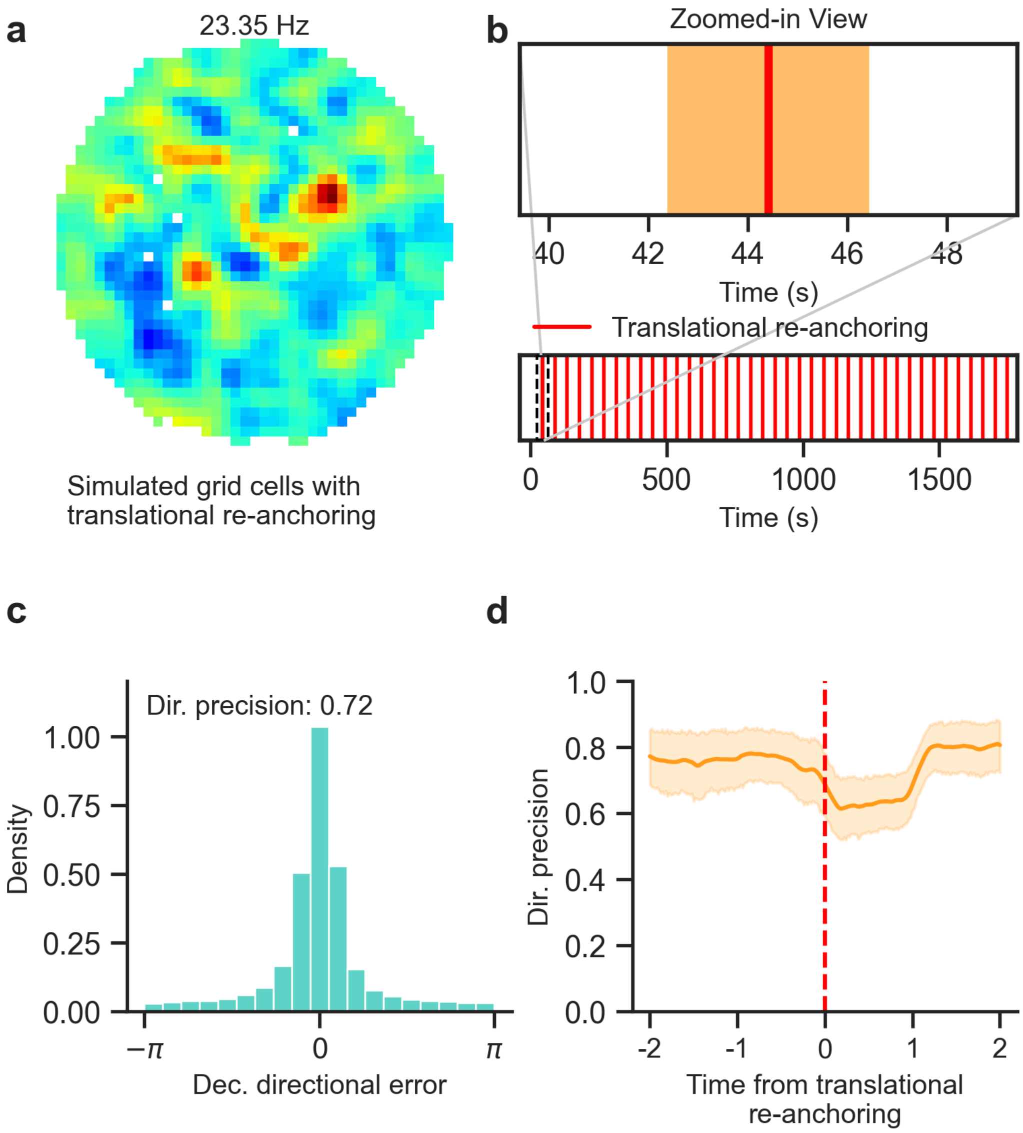

Figure S9: Simulation indicates that directional precision decreases at the moment of translational re-anchoring.

a, Firing rate map of a simulated grid cell that underwent 40 translational re-anchoring events. **b**, For each translational re-anchoring, a 4-second window centered on the event is used. **c**, Distribution of decoded directional error and directional precision (MVL) of the simulation. **d**, Directional precision of the moving average (bin = 1s) of the decoded direction error distribution within the 4-second window across all 40 re-anchoring events. The red dashed line indicates the moment of translational re-anchoring in the simulation.

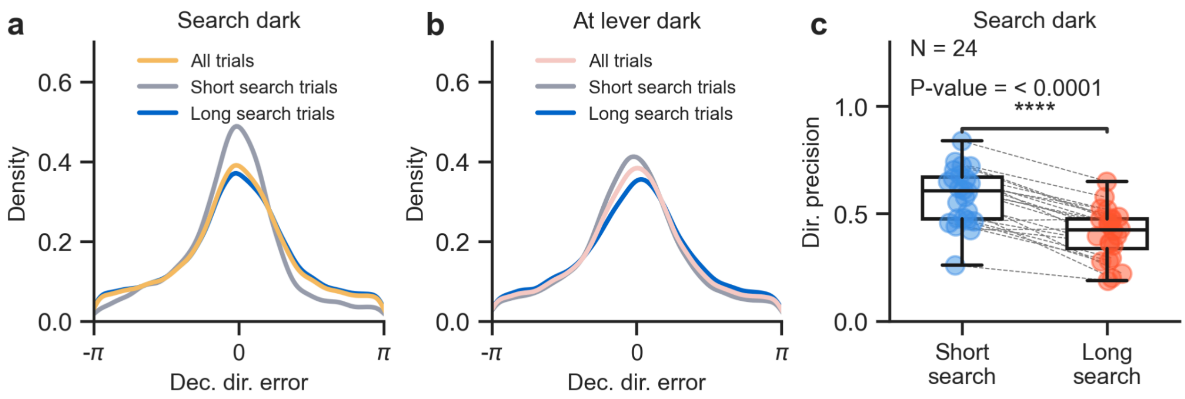

Figure S10. Long search trials show lower directional precision in darkness.
a, Distribution of decoded directional error for trials in search during dark trials. Data are categorized into all trials, short search trials, and long search trials. **b**, Distribution of decoded directional error for trials at lever during dark trials. Data are categorized into all trials, short search trials, and long search trials. **c**, Directional precision of the model during the search phase in short or long search paths (N = 24 sessions, Wilcoxon sign-rank test, $P = 5.960 \times 10^{-7}$).

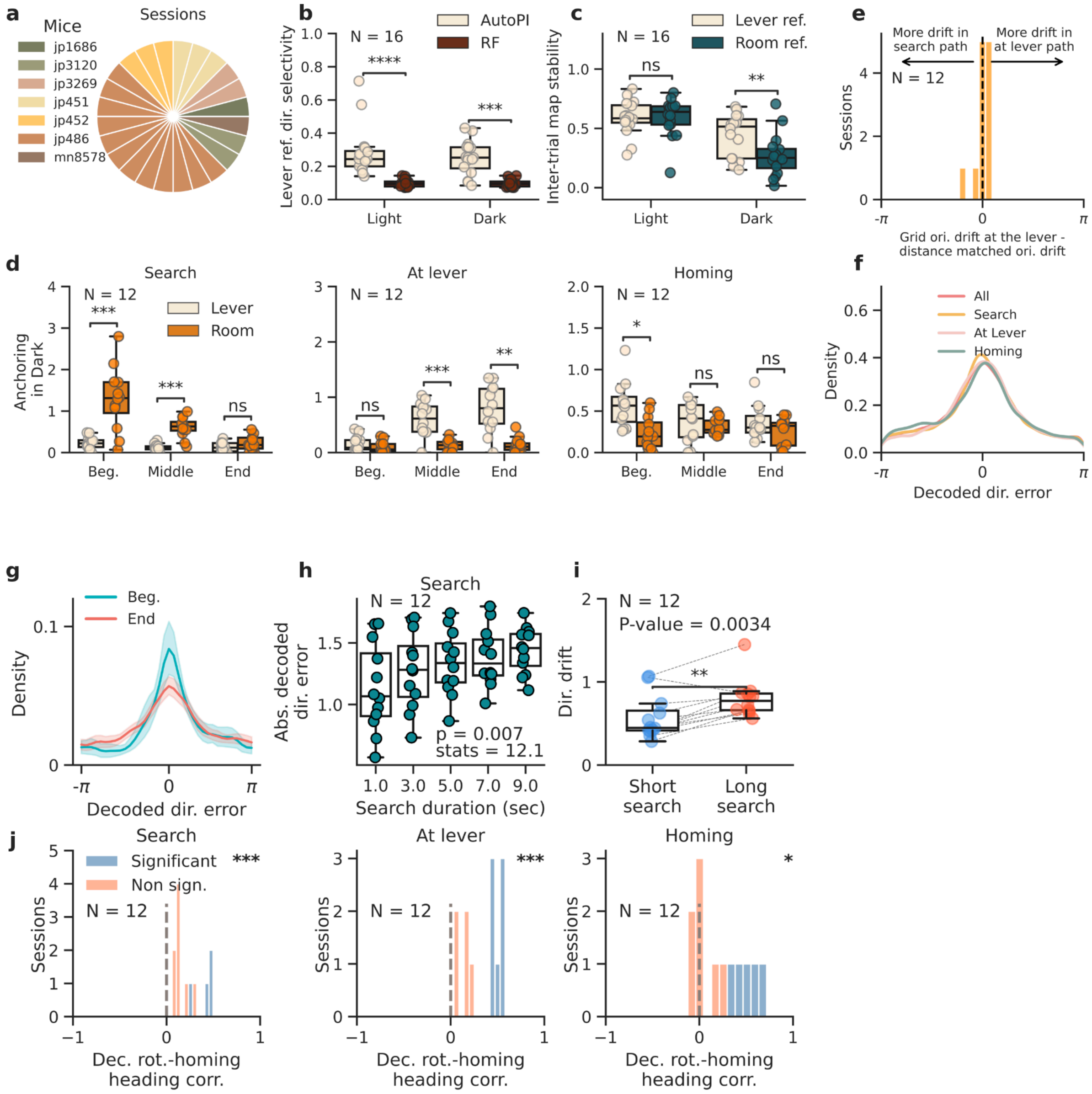

Figure S11: Main conclusions of the manuscript hold true excluding the mouse with the most grid cells.
a, Proportion of different mice in the 24 sessions we used for model reconstruction. The rest of the figures are recreated figures from the previous figure panels excluding jp486, the mouse with the most number of grid cells. **b-f,** Recreated figures from Fig 6. **g-i,** Recreated figures from Fig 7. **j,** Recreated figures from Fig 8.

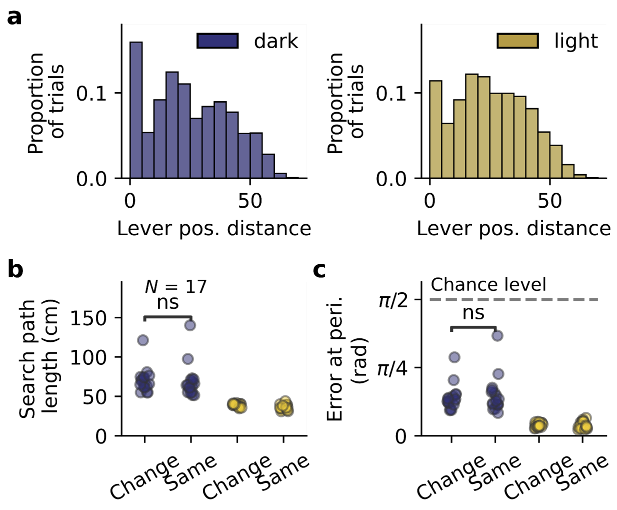

Figure S12: Unchanged lever position does not influence search or homing behavior.

a, Histogram of the Euclidean distance in lever position between consecutive trials for light and dark trials. **b**, Search path length plotted separately for dark and light trials with and without change in lever position before the trial. Change in lever position was defined as a Euclidean distance between the current lever position and the lever position in the previous trials of at least 5 cm (the first bin in the histograms in a). Wilcoxon sign-rank test dark - change vs dark - same: statistic = 47.0, P = 0.17, dark - same vs light - same: statistic: 0.0, P = 1.53 x 10^{-5} c, Similar as in b, but the error at periphery is shown. Wilcoxon sign-rank test dark - change vs dark - same: statistic = 67.0, P = 0.68, dark - same vs light - same: statistic: 0.0, P = 1.53 x 10^{-5}.

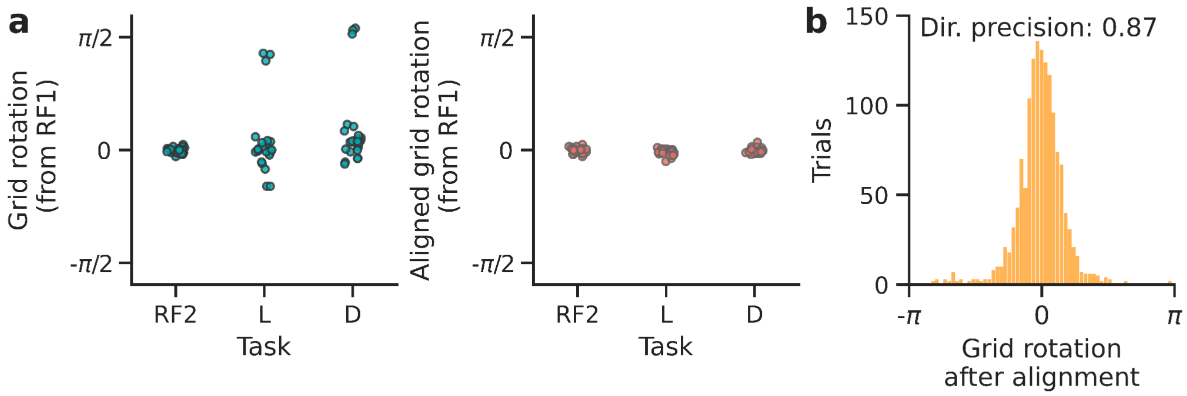

Figure S13: Grid rotation before and after alignment

a, Rotation of the grid representation during the second random foraging (RF2) and light (L) and dark (D) trials on the AutoPI task. The left panel is the same as Fig. 5j. The right panel is the grid rotation after aligning the axes of the torus in the AutoPI task light and dark trials to axes in random foraging 2. **b**, Distribution of grid rotational drift per trial for dark trials after session-wise alignment. The average absolute grid rotation is radian 0.39 (22.3 degrees) (N = 1434 trials across 24 sessions).

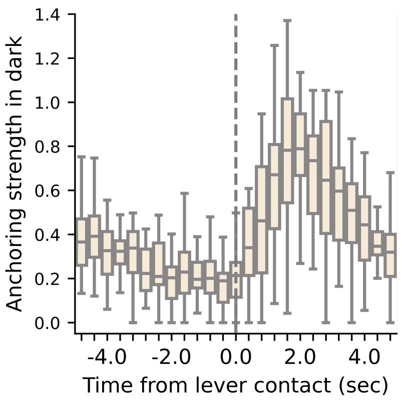

Figure S14: Time dynamics of anchoring to the lever

Anchoring strength to the lever reference frame during dark trials as a function of time from finding the lever. All trials were aligned to lever contact and binned into intervals of 0.4 sec (N = 24 sessions).

Mouse	Session	Grid cell number	Directional precision
jp1686	#1	8	0.543554
jp3120	#1	14	0.576707
jp3120	#2	14	0.733643
jp3269	#1	11	0.53387
jp3269	#2	25	0.736536
jp451	#1	12	0.615106
jp451	#2	13	0.651492
jp451	#3	14	0.641627
jp452	#1	8	0.523374
jp452	#2	22	0.665026
jp452	#3	17	0.77846
jp486	#1	38	0.780456
jp486	#2	11	0.657502
jp486	#3	11	0.577752
jp486	#4	24	0.75043
jp486	#5	13	0.582936
jp486	#6	16	0.530921
jp486	#7	14	0.583552
jp486	#8	14	0.581735
jp486	#9	19	0.775999
jp486	#10	18	0.743145
jp486	#11	38	0.780031
jp486	#12	13	0.52637
mn8578	#1	10	0.595914

Supplementary Table 1. Mice and grid cell numbers of the 24 sessions used for reconstruction.
