# Supplementary material for: Grid cells accurately track movement during path integration-based navigation despite switching reference frames
Source: Nat Neurosci. 2025 Sep 10;28(10):2092–105. doi: 10.1038/s41593-025-02054-6 (PMC12497648; doi:10.1038/s41593-025-02054-6)
Supplement: Supplementary file 2 — Reporting Summary [file 41593_2025_2054_MOESM2_ESM.pdf]

nature portfolio

Corresponding author(s): Dr Hannah Monyer, Dr Kevin Allen

Last updated by author(s): Jun 23, 2025

Reporting Summary

Nature Portfolio wishes to improve the reproducibility of the work that we publish. This form provides structure for consistency and transparency in reporting. For further information on Nature Portfolio policies, see our Editorial Policies and the Editorial Policy Checklist.

Statistics

For all statistical analyses, confirm that the following items are present in the figure legend, table legend, main text, or Methods section.

n/a	Confirmed	
☐	☒	The exact sample size (*n*) for each experimental group/condition, given as a discrete number and unit of measurement
☐	☒	A statement on whether measurements were taken from distinct samples or whether the same sample was measured repeatedly
☐	☒	The statistical test(s) used AND whether they are one- or two-sided<br>*Only common tests should be described solely by name; describe more complex techniques in the Methods section.*
☐	☒	A description of all covariates tested
☐	☒	A description of any assumptions or corrections, such as tests of normality and adjustment for multiple comparisons
☐	☒	A full description of the statistical parameters including central tendency (e.g. means) or other basic estimates (e.g. regression coefficient) AND variation (e.g. standard deviation) or associated estimates of uncertainty (e.g. confidence intervals)
☐	☒	For null hypothesis testing, the test statistic (e.g. *F*, *t*, *r*) with confidence intervals, effect sizes, degrees of freedom and *P* value noted<br>*Give P values as exact values whenever suitable.*
☐	☒	For Bayesian analysis, information on the choice of priors and Markov chain Monte Carlo settings
☐	☒	For hierarchical and complex designs, identification of the appropriate level for tests and full reporting of outcomes
☐	☒	Estimates of effect sizes (e.g. Cohen's *d*, Pearson's *r*), indicating how they were calculated

Our web collection on statistics for biologists contains articles on many of the points above.

Software and code

Policy information about availability of computer code

Data collection	https://github.com/kevin-allen/ktan 0.1;<br>https://github.com/jamesjun/Kilosort2 default;<br>https://github.com/cortex-lab/phy 2.0a1;<br>https://github.com/kevin-allen/positrack2 0.0.1;
Data analysis	https://github.com/kevin-allen/autopipy.git,<br>https://github.com/kevin-allen/spikeA.git,<br>https://github.com/PJJ19/Peng_et.al_2025_noInt,<br>https://github.com/PJJ19/autopi_grid_cell_decoding,<br>Python 3.11.5<br>scipy 1.11.3<br>scikit-learn 1.5.0<br>seaborn 0.12.2<br>statannotations 0.6.0<br>deeplabcut 2.2.3<br>pytorch 2.3.1<br>numpy 1.26.4<br>pandas 1.5.3<br>unetTracker 0.0.1

For manuscripts utilizing custom algorithms or software that are central to the research but not yet described in published literature, software must be made available to editors and reviewers. We strongly encourage code deposition in a community repository (e.g. GitHub). See the Nature Portfolio guidelines for submitting code & software for further information.

Data

Policy information about availability of data

All manuscripts must include a data availability statement. This statement should provide the following information, where applicable:

- Accession codes, unique identifiers, or web links for publicly available datasets
- A description of any restrictions on data availability
- For clinical datasets or third party data, please ensure that the statement adheres to our policy

https://doi.org/10.5061/dryad.f7m0cfz80

Research involving human participants, their data, or biological material

Policy information about studies with human participants or human data. See also policy information about sex, gender (identity/presentation), and sexual orientation and race, ethnicity and racism.

Reporting on sex and gender	NA
Reporting on race, ethnicity, or other socially relevant groupings	NA
Population characteristics	NA
Recruitment	NA
Ethics oversight	NA

Note that full information on the approval of the study protocol must also be provided in the manuscript.

Field-specific reporting

Please select the one below that is the best fit for your research. If you are not sure, read the appropriate sections before making your selection.

☒ Life sciences ☐ Behavioural & social sciences ☐ Ecological, evolutionary & environmental sciences

For a reference copy of the document with all sections, see nature.com/documents/nr-reporting-summary-flat.pdf

Life sciences study design

All studies must disclose on these points even when the disclosure is negative.

Sample size	We had 17 mice in the recording task. No estimate of statistical power was performed. The sample size was determined based on previous studies. Gil, M., et el. Nat Neurosci (2018); M Najafian Jazi., et el. Nat Comm(2023)
Data exclusions	Grid cell movement decoding was attempted when at least 5 co-modular grid cells were recorded (49 sessions from 11 mice) because fewer grid cells produced decoding results near chance level. The model was trained with the data from the first random foraging trial and tested on the data from the second random foraging trial. Only sessions in which the directional precision on the test set was above 0.5 were included for subsequent decoding analysis (24 sessions). Because animals tend to move along the arena borders during random foraging, we excluded border data when testing model performance to ensure that decoding accuracy remained reliable for behaviors on the arena center. Additionally, we included spiking activity in the model analysis only when the animal was moving on the arena (>10cm/s). Exclusion criteria were established during the RNN model development but prior to data analysis in the AutoPI task.
Replication	To perform replication we need to sacrifice more mice, which is ethically not correct.
Randomization	We recorded from grid cells in the medial entorhinal cortex when the animal performed the AutoPI task. Comparisons were made within the same subject or session across different behavioral conditions, randomization is not relevant in our study. For randomization of the behavioral task, the arena was rotated after each trial to one of eight possible angles (multiple of 45°). The lever moved to a new random location on the arena every fourth trial.
Blinding	Not relevant as these experiments were not a case-controlled study.

Reporting for specific materials, systems and methods

We require information from authors about some types of materials, experimental systems and methods used in many studies. Here, indicate whether each material, system or method listed is relevant to your study. If you are not sure if a list item applies to your research, read the appropriate section before selecting a response.

Materials & experimental systems

n/a	Involved in the study	
☒	☐	Antibodies
☒	☐	Eukaryotic cell lines
☒	☐	Palaeontology and archaeology
☐	☒	Animals and other organisms
☒	☐	Clinical data
☒	☐	Dual use research of concern
☒	☐	Plants

Methods

n/a	Involved in the study	
☒	☐	ChIP-seq
☒	☐	Flow cytometry
☒	☐	MRI-based neuroimaging

Animals and other research organisms

Policy information about studies involving animals; ARRIVE guidelines recommended for reporting animal research, and Sex and Gender in Research

Laboratory animals	C57BL6/N Mice, age 5-9 month old; The mice were single-housed in 26 x 20 x 14 cm plexiglass cages; Room temperature around 23.2°C, room humidity around 52.9%.
Wild animals	None
Reporting on sex	17 Male mice
Field-collected samples	None
Ethics oversight	All procedures followed the European Council Directive (86/609/EEC) and were approved by the Governmental Supervisory Panel on Animal Experiments of Baden Württemberg in Karlsruhe (G-236/20).

Note that full information on the approval of the study protocol must also be provided in the manuscript.

Plants

Seed stocks	*Report on the source of all seed stocks or other plant material used. If applicable, state the seed stock centre and catalogue number. If plant specimens were collected from the field, describe the collection location, date and sampling procedures.*
Novel plant genotypes	*Describe the methods by which all novel plant genotypes were produced. This includes those generated by transgenic approaches, gene editing, chemical/radiation-based mutagenesis and hybridization. For transgenic lines, describe the transformation method, the number of independent lines analyzed and the generation upon which experiments were performed. For gene-edited lines, describe the editor used, the endogenous sequence targeted for editing, the targeting guide RNA sequence (if applicable) and how the editor was applied.*
Authentication	*Describe any authentication procedures for each seed stock used or novel genotype generated. Describe any experiments used to assess the effect of a mutation and, where applicable, how potential secondary effects (e.g. second site T-DNA insertions, mosiacism, off-target gene editing) were examined.*
